# Proteomics approach to discovering non-invasive diagnostic biomarkers and understanding the pathogenesis of endometriosis: a systematic review and meta-analysis

**DOI:** 10.1186/s12967-024-05474-3

**Published:** 2024-07-26

**Authors:** Getnet Gedefaw Azeze, Ling Wu, Bekalu Kassie Alemu, Wing Fong Lee, Linda Wen Ying Fung, Eva Chun Wai Cheung, Tao Zhang, Chi Chiu Wang

**Affiliations:** 1 Department of Obstetrics and Gynaecology, Faculty of Medicine, Prince of Wales Hospital, The Chinese University of Hong Kong, Shatin, Hong Kong SAR; 2 Department of Midwifery, College of Medicine and Health Sciences, Injibara University, Injibara, Ethiopia; 3 Department of Midwifery, College of Medicine and Health Sciences, Debre Markos University, Debre Markos, Ethiopia; 4 School of Biomedical Sciences; Li Ka Shing Institute of Health Sciences; Chinese University of Hong Kong – Sichuan University Joint Laboratory in Reproductive Medicine, The Chinese University of Hong Kong, Shatin, Hong Kong SAR

**Keywords:** Proteomics, Endometriosis, Meta-analysis, Blood, Urine, Cervical mucus, Biomarker

## Abstract

**Background:**

Endometriosis is one of the most common gynaecological diseases, yet it lacks efficient biomarkers for early detection and unravels disease mechanisms. Proteomic profiling has revealed diverse patterns of protein changes in various clinical samples. Integrating and systematically analysing proteomics data can facilitate the development of biomarkers, expediting diagnosis and providing insights for potential clinical and therapeutic applications. Hence, this systematic review and meta-analysis aimed to explore potential non-invasive diagnostic biomarkers in various biological samples and therapeutic targets for endometriosis.

**Methods:**

Online databases, including Scopus, PubMed, Web of Science, MEDLINE, Embase via Ovid, and Google Scholar, were searched using MeSH terms. Two independent authors screened the articles, extracted the data, and assessed the methodological quality of the included studies. GO and KEGG analyses were performed to identify the pathways that were significantly enriched. Protein‑protein interaction and hub gene selection analyses were also conducted to identify biomarker networks for endometriosis.

**Results:**

Twenty-six observational studies with a total of 2,486 participants were included. A total of 644 differentially expressed proteins (180 upregulated and 464 downregulated) were identified from 9 studies. Proteins in peripheral blood exhibited a sensitivity and specificity of 38-100% and 59-99%, respectively, for detecting endometriosis, while proteins in urine had a sensitivity of 58-91% and specificity of 76-93%. Alpha-1-antitrypsin, albumin, and vitamin D binding proteins were significantly DEPs in both serum and urine. Complement C3 is commonly expressed in serum, menstrual blood, and cervical mucus. Additionally, S100-A8 is commonly expressed in both menstrual blood and cervical mucus. Haptoglobin is commonly detected in both serum and plasma, whereas cathepsin G is found in urine, serum, and plasma. GO and KEGG enrichment analyses revealed that proteoglycans in cancer pathways, which regulate cell-to-cell interactions, modulate the extracellular matrix, and promote the proliferation and invasion of endometrial cells, are commonly enriched in serum and urine.

**Conclusion:**

This comprehensive study revealed potential proteomes that were significantly differentially expressed in women with endometriosis utilizing various non-invasive clinical samples. Exploring common differentially expressed proteins in various biological samples provides insights into the diagnosis and pathophysiology of endometriosis, as well as potential clinical and therapeutic applications.

**Graphical abstract:**

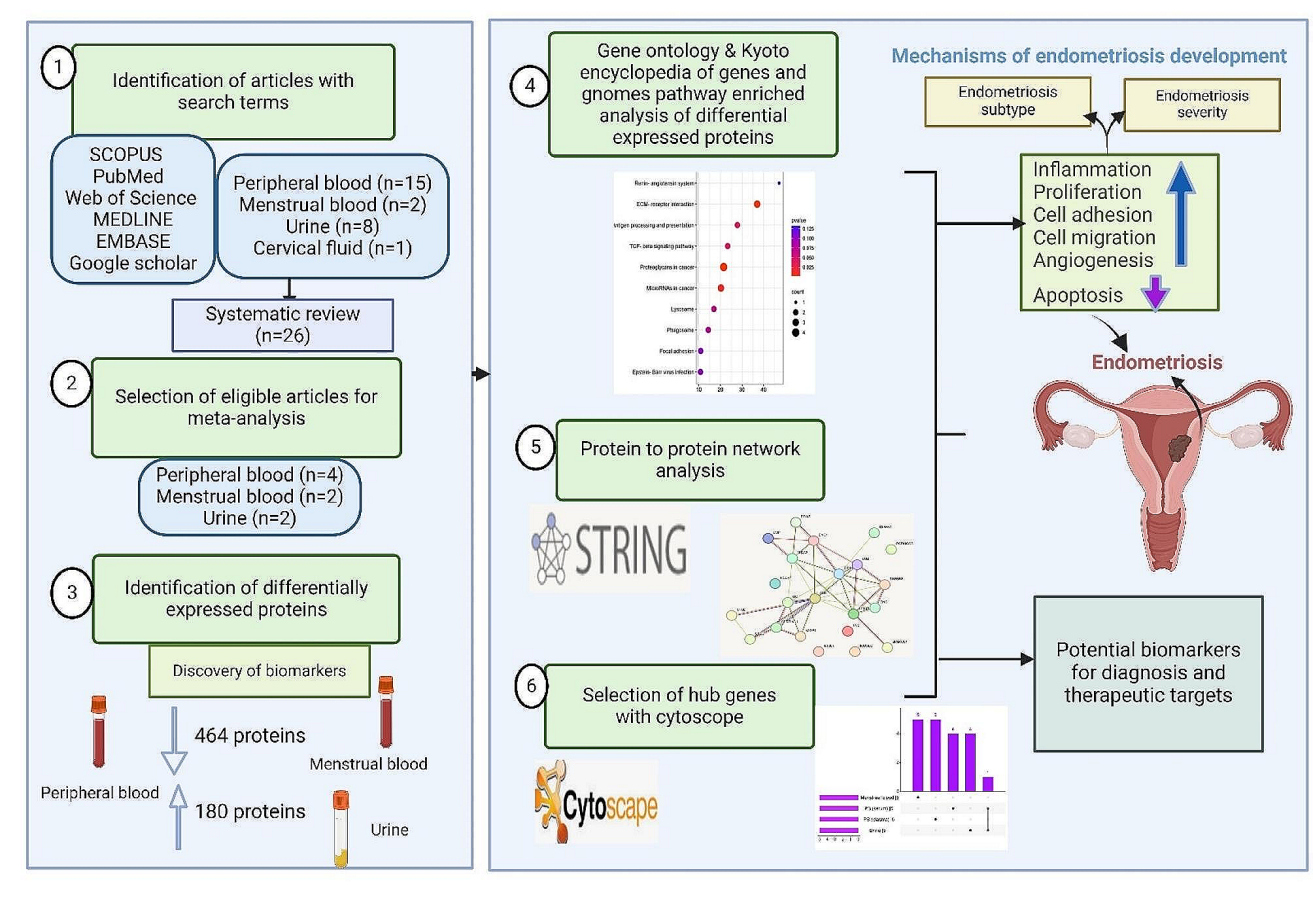

**Supplementary Information:**

The online version contains supplementary material available at 10.1186/s12967-024-05474-3.

## Background

Endometriosis is characterized by the development of endometrium-like tissue and/or stroma outside the endometrium and myometrium [1, 2]. It is a chronic inflammatory disease that affects more than 170 million women worldwide, predominantly women of reproductive age, with a wide range of clinical symptoms, including dysmenorrhea, dyspareunia, dyschezia, dysuria, chronic pelvic pain, and infertility, affecting women’s health from the time of menarche to menopause, regardless of their ethnicity or social status [1, 3].

In clinical settings, the gold standard diagnostic method for confirming endometriosis is laparoscopy, a minimally invasive surgical procedure that involves inserting an imaging tube through a small incision in the abdomen [4]. Although laparoscopy is effective and the gold standard, it has potential complications, requires general anaesthesia, and demands advanced surgical skills [5–7]. Moreover, it is not always available or accessible, particularly in low- and middle-income countries where healthcare facilities and resources are lacking [5]. Ultrasound is the first-line non-invasive diagnostic method for detecting endometriosis [8]. It has been widely used to enhance the diagnosis and identification of endometriomas and nodules in adjacent structures of the pelvis but lacks both sensitivity and specificity for ruling out peritoneal endometriosis, endometriosis-associated adhesions, and deep infiltrating endometriosis [9, 10]. Imaging techniques such as transvaginal ultrasound (TVS), transrectal ultrasound (TRS), and magnetic resonance imaging (MRI) can bridge the gap between clinical and surgical diagnosis by providing a non-invasive visual diagnosis that can be achieved more quickly, safely, and accessibly than surgery. However, different studies have reported wide variation in diagnostic accuracy between MRI and TVS, mainly due to the variability of techniques, examiners’ experience, and anatomic locations of the lesions/subtypes of the disease [11]. Given these challenges, non-invasive diagnostic approaches for endometriosis are urgently needed.

While various non-invasive diagnostic modalities involving blood, cervicovaginal fluid, and urine have been proposed, a definitive diagnostic biomarker for endometriosis remains elusive. Despite extensive research into blood and urine tests and the investigation of altered levels of cytokines, angiogenic factors, and growth factors, none of these biomarkers have been used to conclusively diagnose endometriosis [12–15]. In addition, numerous studies have demonstrated that nanoparticles, which are materials with dimensions smaller than 100 nanometers, hold promise for improving diagnostic and imaging techniques for non-invasive detection, understanding target signalling pathways, and identifying therapeutic options for diverse diseases [16–19]. Notably, nanoparticles can serve as carriers for transporting anti-inflammatory, antioxidant, anti-angiogenic, or immunomodulatory molecules to specific locations [20–23], owing to their low toxicity, high stability, and capacity for conjugating with various biomolecules [21, 24, 25]. Moreover, nanotechnology may offer a promising non-invasive diagnostic method for detecting endometriosis by identifying specific biomarkers, such as proteins or genetic materials [26]. Although studies have shown that CA 19 − 9 and CA-125 have been detected in blood using immunochemical sensing [27, 28], the recognition of iron oxide nanoparticles as contrast agents for magnetic resonance imaging [26, 29], and the investigation of gold nanorods and carbon nanotubes as photoacoustic imaging agents for visualizing endometriosis lesions in vivo [26, 30]. However, it is important to note that none of the biomarkers/methods have been clinically proven biomarkers for endometriosis detection. Among all techniques, proteomic approaches are essential for identifying biomarkers by characterizing the protein content of biological samples [31]. These approaches enable proteome profiling, comparative expression analysis of proteins in various biological samples, identification of posttranslational modifications, and identification of protein–protein interactions. Notably, proteomic analysis is invaluable because proteins, unlike DNA or RNA, directly mediate cellular functions and disease mechanisms [32, 33]. Mass spectrometry (MS) proteomic methods have appeared to be powerful platforms for discovering novel and potential diagnostic and prognostic biomarkers for various diseases. MS-based approaches are substantially helpful for consistently identifying proteins with high diagnostic accuracy for endometriosis [34]. Furthermore, proteomics studies offer functional insights into expressed proteins and significantly enriched pathways, providing valuable information for understanding the pathogenesis of this disease.

Our hypothesis is that biomarkers of endometriosis commonly found in various biological samples may have substantial significance and have a direct impact on the development and progression of endometriosis. Therefore, our aim is to gain a thorough understanding of the diagnosis, pathogenesis, and possible therapeutic approaches for endometriosis utilizing diverse clinical samples, which could ultimately result in improved patient outcomes and quality of care. Hence, this systematic review aims to assess the utility of proteomic (MS-targeted) analysis for biomarker discovery and navigate the pathogenesis of endometriosis development. Additionally, this study explored the sensitivity and specificity of expressed proteins as promising biomarkers for detecting endometriosis. Moreover, this study involved mass spectrometry-based diagnostic testing for endometriosis and a comprehensive understanding of the pathogenesis of endometriosis in various non-invasive biological samples, including peripheral blood, cervical mucus, menstrual blood, and urine. Remarkably, this study examined commonly enriched pathways associated with disease conditions to better understand the mechanism of disease development.

## Methods

### Protocol registration

Following the PRISMA 2020 checklist, we conducted a systematic review and registered the protocol with PROSPERO (registration ID: CRD42023397217).

### Study search strategy

Searches were performed in the following databases: PubMed, EMBASE through OVID, Google Scholar, Scopus, and Web of Science. The following terms were used in the search strategy, with alternatives as shown using Boolean operators: “mass spectrometry” AND (“diagnostic” OR “test”) AND (“endometriosis” OR “endometrioma”) & (‘’proteomics’’ OR’’ proteome’’ AND (‘’endometriosis’’ OR’’ endometrioma”).

In addition, manual searches were performed for the reference lists of all studies identified by the search strategy described above. Web sources and databases were searched for published articles and preprint research papers written in the English language up to January 31, 2024.

### Study participants

The participants in the study were reproductive-aged women who underwent laparoscopy or abdominal surgery for one of the following reasons: pelvic pain, infertility, dysmenorrhea, abnormal pelvic examination, or a combination of the aforementioned conditions, an ovarian mass regardless of symptoms, or other pelvic pathologies. Only confirmed cases with laparoscopy and/or histology data were included in the review after surgery, while women with confirmed benign pelvic pathologies, such as uterine fibroids, ovarian cysts, unexplained infertility, and fertile healthy women were considered as controls.

### Study selection

From the initial 2,273 retrieved articles, we included 22 case-control, 2 cross-sectional, and 2 prospective cohort studies that met our eligibility criteria. Laparoscopy or laparotomy with or without histological confirmation and mass spectrometry techniques were used as reference standards and index tests, respectively.

### Inclusion criteria

In this study, women with a confirmed diagnosis of endometriosis, either combined with one phenotype (I) ovarian endometriosis, (II) deep pelvic infiltrating endometriosis (DIE), and (III) peritoneal endometriosis, were enrolled as cases, whereas women with benign uterine conditions such as uterine fibroids and ovarian cysts and healthy women (self-declaration) were enrolled as controls. All observational studies, such as cohort, cross-sectional, and case-control studies, that were published exclusively in the English language were considered for inclusion.

### Exclusion criteria

Endometriosis with other coincidental pelvic pathologies, such as pelvic malignancy, adenomyosis, polycystic ovarian syndrome (PCOS), and pelvic inflammatory disease (PID), studies conducted with approaches other than mass spectrometry-based series, proteomics studies with invasive sources of samples, such as peritoneal fluid, endometrial biopsy, follicular fluid, and endometrial fluid, and studies reporting proteins with other index tests, such as enzyme-linked immunosorbent assay (ELISA), polymerase chain reaction (PCR/qPCR), and western blot, were excluded from the study. Additionally, case reports or series, articles without full text and abstracts, duplicated studies, anonymous reports, editorial reports, reviews, perspectives, and book sections or chapters were also excluded.

### Data extraction

The authors’ names, year of study, country, diagnostic criteria for endometriosis, type of sample, protein alterations, menstrual phase, proteomics platform, sensitivity, and specificity of biomarkers with a molecular weight of m/z were extracted from each article (Table 1). In addition, for the bioinformatics analysis, the protein ID (UniProt), protein accession, and fold change (up- and downregulated) were extracted. Moreover, the protein lists from the 8 articles were extracted, including the identification codes and the level of regulation (up/downregulated). The UniProt website (https://www.uniprot.org/) was used to standardize the protein identification codes. Subsequently, a comparison was conducted on the significantly differentially expressed proteins extracted from the 8 papers to identify consistent proteins. Studies reporting the *p* value (*p* < 0.05) and fold change (FC) of differentially expressed proteins were included in the meta-analysis.

**Table 1:** Proteomics studies of endometriosis

Author (year) & country	Study design	Age of the patient/control (mean ± SD/SEM, median with IQR)	Diagnosis criteria	Type of Sample	Menstrual cycle	Number of subjects (case/control)	Proteomics platform/surface	Main findings: Proteins (m/z), in Da.	Sensitivity/specificity (%)
Zhang et al. 2023 [93], China	Case control	35.375 ± 4.069/34 ± 4.721#	Histology	menstrual blood	menstrual phase	8/8	LC‒MS/MS	95 DEPs (64↑ and 31↓)	NR
Višnić et al., 2023 [94], Croatia	Case control	NA	Histology	urine	Proliferative phase	16/16	LC‒MS/MS	17 DEPs (15↑ and 2↓)	NR
Sasamoto et al. 2022 [62], USA	Cross sectional	18/22#	Laparoscopy	plasma	NR	142/78	SOMAscan	63 proteins associated with endometriosis (36↑, 27↓)	NR
Penariol et al. 2022 [95], Brazil	Case control	18–40*	Laparoscopy with histology	menstrual blood	menstrual phase	10/9	UHPLC-MS/MS	1373 proteins expressed	NR
Giuseppe et al. 2020 [96], Italy	Case control	30–40**	Laparoscopy with histology	urine	NR	8/5	LC‒MS/MS	11 proteins ↑ and one ↓ in endometriosis	NR
Chen et al. 2019 [41], China	Case control	35.8 ± 8.3/38.4#	Laparoscopy with histology	urine	NR	25/72	LC‒MS/MS	Histone 4	70/80
Manousopoulou et al. 2018 [97], UK	Case control	32.5 ± 4.5/31.8 ± 4.7	Laparoscopy	serum	proliferative phase	4/4	LC‒MS/MS	404 (DEPs) identified in endometriosis	NR
Grande et al. 2017 [98], Italy	Case control	30–40**	Laparoscopy with histology	cervical mucus	periovulatory phase	10/10	LC‒MS/MS	6 and 9 proteins were quantitatively ↑ and ↓ in endometriosis	NR
Zhao et al. 2015 [99], China	Case control	37.8/38.9 #	Laparoscopy with histology	serum	NR	50/40	2DE with MALDI-TOF/MS	5 proteins, with m/z of 4210, 5904, and 2660	96.67/100
Dutta et al. 2014 [100], India	Case control	28.18 /28.49#	Laparoscopy	serum	NR	547/79	2D-DIGE combined with MALDI-TOF/TOF-MS	25 DEPs identified & alpha-1B-glycoprotein (A1BG) identified as a promising diagnostic biomarker	NR
Hwang et al. 2014 [47], South Korea	Case control	NA	Histology	plasma	proliferative phase	15/15	2DE, ESI-Q-TOF/MS	C4A and α-2 M protein expression ↑in endometriosis Hp, ApoL-1, and LRG ↓in endometriosis	NR
Wang et al. 2014 [101], China	Case control	30.5 + 3.4/31.5 + 4.2#	Laparoscopy	urine	proliferative & secretory phases	60/62	MALDI-TOF/LC‒MS/MS	5 proteins, with m/z of 433.9, 1599.4, 2085.6, 6798.0 and 3217.2.	90.9/92.9
Williams et al. 2014 [102], USA	Case control	NA	Laparoscopy/Laparotomy	urine	NR	17/44	iTRAQ & 2D LC‒MS/MS	1025 DEPs are identified. Uromodulin, serum albumin, keratins, various immunoglobulins, actin, and collagen were among the proteins detected in the majority of samples	NR
Zhang et al. 2013 [103], China	Case control	24–38**	Laparotomy	serum	NR	32/34	MALDI-TOF-MS	1 protein, with m/z of 4180	100/90
Fassbender et al. 2012 [104], Belgium	Case control	31.44 + 4.24/ 32.32 + 0.19#	Laparoscopy with histology	plasma	menstrual, secretory & proliferative phases	165/89	SELDI-TOF-MS (CM10, SPA)	5 proteins, with m/z of 9,926.31, 10,072.2, 6,753.04, 4,302.67, 9,328.49	38/85
								5 proteins, with m/z of 1,366.3, 5,712.69, 10,070.7, 3,017.68, 3,824.44	53/82
								5 proteins, with m/z of 2,831.02,7,554.66, 4,241.29, 2,953.25, and 9,927.73	66/99
Faserl et al. 2011 [38], Austria	Cross sectional	NA	Laparoscopy	serum	NR	56/20	2DE with MALDI-TOF/MS	vitamin D-binding protein was higher in all endometrioses by 3 folds	NR
Cho et al. 2011 [37], Korea	Case control	34.2 ± 6.88 /32.7 ± 10.26#	Laparoscopy/histology	urine	proliferative & secretory phases	57/38	2DE with LC‒MS/MS	VDBP protein, with m/z of 52,930.	58/76
El-Kasti et al. 2011 [105], UK	Prospective randomized pilot study	35#	Laparoscopy	urine	periovulatory & secretory phase	23/16	MALDI-TOF/MS	1 during the periovulatory phase and 1 during the luteal phase with m/z of 1,767.1 and 1,824.3, respectively (control vs. moderate/severe Endometriosis)	75/75 and 84.6/71.4
								2 peptide markers (1 during the periovulatory phase) 1 during the luteal phase with m/z of 3,280.9 and 1,933.8, respectively (minimal/mild vs. moderate/severe Endometriosis)	81.8/75 and 87.5/75
Seeber et al. 2010 [106], USA	Case control	NA	Laparoscopy	serum	NR	61/78	SELD-TOF-MS/CM10	6 proteins, with m/z of 1629 3047, 3526, 3774, 5046 & 5068	66/99
Tokushige et al. 2010 [107], Australia	Case control	32.8/38.6#	Laparoscopy with histology	urine	proliferative & secretory phase	11/6	2DE with MALDI-TOF/MS	133 DEPs were significantly different between women with and without endometriosis. Cytokeratin-1 is highly ↑ in endometriosis	NR
Jing et al. 2009 [108], Japan	Case control	22–48 **	Laparoscopy with histology	serum	NA	30/31	SELDI-TOF-MS/IMAC30	2 proteins, with m/z of 5,830 Da and 8,865	86.67/96.77
Zhang et al. 2009 [109], China	Case control	NA	Histology	serum	NA	36/24	SELDI-TOF-MS/CM10	3 proteins, with m/z of 4974, 5813 and 4290	91.7/95.8
Liu et 2009 [110], China	Case control	NA	Laparoscopy	plasma	NR	36/35	SELDI-TOF-MS	3 proteins, with m/z of 3956, 11 710, and 6 986	92/83
Wolfler et al. 2009 [111], Germany	Prospective cohort	32.5/31.9#	Laparoscopy with histology	serum	proliferative & secretory phases	51/39	SELDI-TOF-MS/Q10	5 proteins, with m/z of 4159, 5264, 5603, 9861 and 10,533	78.4/59
Wang et al. 2008 [112], China	Case control	36/38*	Laparoscopy with histology	Serum	NR	36/30	SELDI-TOF-MS/H4	5 proteins, with m/z of 8142, 5640, 5847, 8940, and 3269	91.7/90.0
Liu et al. 2007 [113], China	Case control	24–46**	Histology	plasma	NR	52/46	SELDI-TOF-MS/WAX2	3 proteins, with m/z of 3,956.83, 11,710.70 & 6,986.45	87.5/85.7

#, Mean with/without standard deviation (SD), **, range; *, median, NR, not reported; DEP, differentially expressed protein; ↑--increased; ↓, decreased; SELDI-TOF-MS, surface-enhanced laser desorption/ionization-time of flight mass spectrometry; 2DE, two-dimensional gel electrophoresis with MALDI-TOF/MS, matrix-assisted laser desorption ionization–time-of-flight mass spectrometry; iTRAQ, isobaric tag for relative and absolute quantitation, LC‒MS, liquid chromatography‒mass spectrometry; 2D DIGE, two-dimensional difference gel electrophoresis, ESI-TOF-MS, electrospray ionization time-of-flight-mass spectrometry; UHPLC, ultrahigh-performance liquid chromatography; m/z, mass-to-charge ratio; DEP, differentially expressed protein

### Risk of bias and applicability

Two authors (GGA & BKA) conducted independent assessments of risks associated with bias and applicability using the Diagnostic Precision Study Quality Assessment Tool (QUADAS-2) for the studies included in the diagnostic accuracy review [35]. Conflicts between the two authors were evaluated and reviewed by a third author (LW). Patient selection, index test, reference standard, and flow and timing were the four domains used to evaluate the risk of bias, whereas patient selection, index test, and reference standard were the domains employed to assess the applicability of each article. The distribution of risk-of-bias and applicability judgments within each bias domain was assessed (Figure S1).

### Identification and enrichment of DEPs

Gene Ontology (GO) and Kyoto Encyclopedia of Genes and Genomes (KEGG) pathway enrichment analyses were performed to elucidate the biological characteristics of the overlapping DEGs via the online tool database for annotation, visualization, and integrated discovery (DAVID) (https://david.ncifcrf.gov/). GO annotation and KEGG pathway analyses were performed with Metascape (https://metascape.org/). Furthermore, a science and research online plot (SRplot) (https://www.bioinformatics.com.cn/en) was used to present the findings. GO and KEGG analyses were performed for each clinical sample separately, such as peripheral blood (serum, plasma), urine, and menstrual blood. DEPs from the supernatant and mesenchymal stem cells derived from menstrual blood were combined and analysed as menstrual blood-expressed proteins. For each given gene list, pathway and process enrichment analyses were carried out with KEGG and GO pathway analyses. Metascape (https://metascape.org/) default parameters: terms with a *p* value < 0.05, a minimum count of 3, and an enrichment factor > 1.5 were deemed significant. Moreover, *p* values are calculated based on the cumulative hypergeometric distribution, and q-values are calculated using the Benjamini‒Hochberg procedure to account for multiple tests [36].

### Protein‒protein interaction (PPI) network construction and analysis

The PPI network was constructed with the STRING (https://string-db.org/) database with a threshold of a combined score > 0.4, and the interaction networks were visualized with Cytoscape (version 3.10.1). In addition, the molecular complex detection (MCODE) plug-in was used to screen strongly interconnected modules in the PPI network with default parameters (degree cut-off = 2, node score cut-off = 0.2, and K-score = 2).

### Hub gene selection and analyses

The Cyto-Hubba plug-in in Cytoscape (version 3.10.1) was used to select hub genes in the PPI network. Based on the evidence in the literature, we selected five of the 12 algorithms in the cyto-Hubba plug-in and took the intersection of the five parameters (degree, edge percolated component, maximum neighborhood component, maximal clique centrality, and eccentricity) to determine the hub genes in each biological sample.

## Results

### Study characteristics

**Fig. 1:**
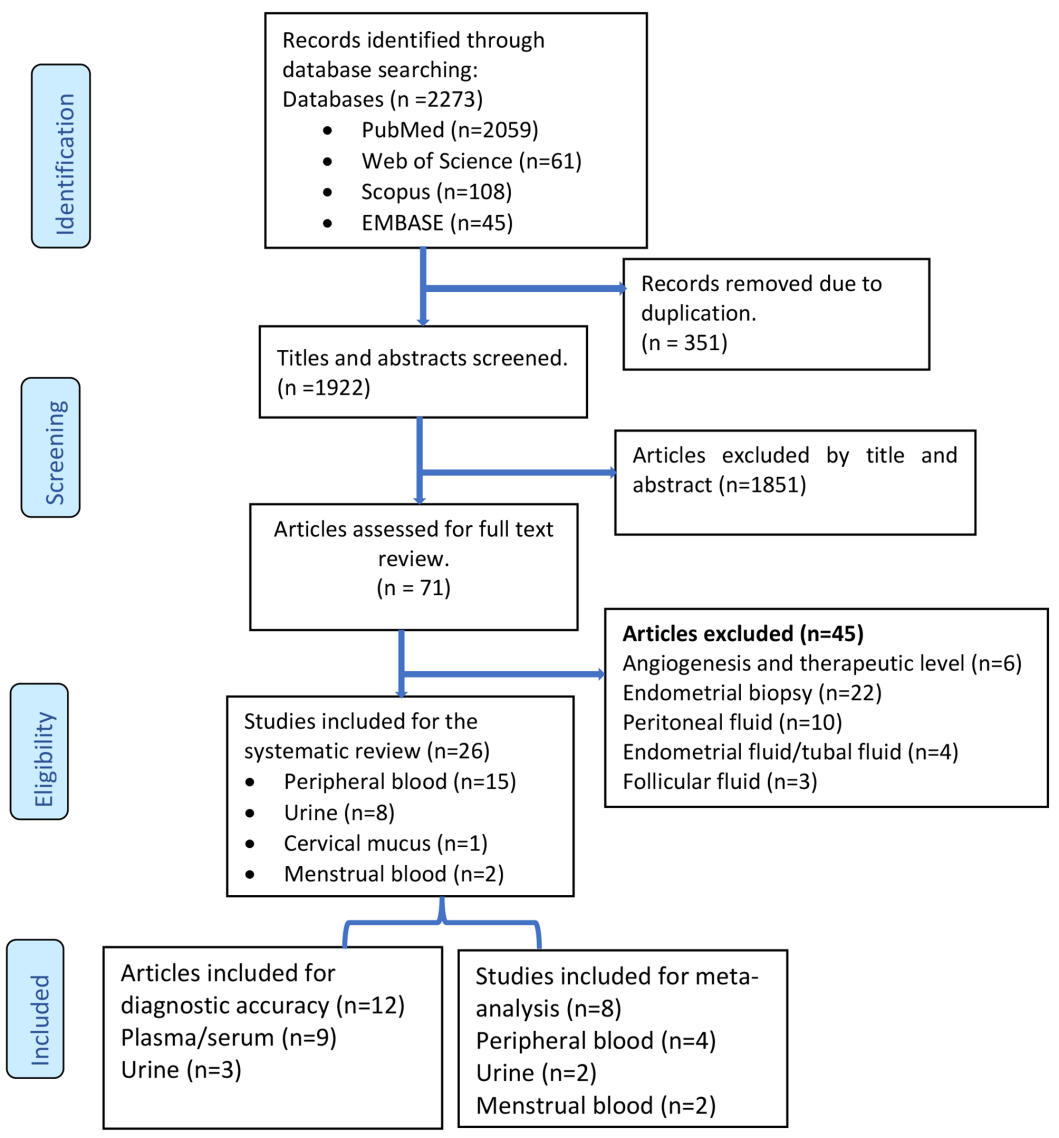
PRISMA flowchart

A total of 2,273 articles were identified from the online databases with the search strategy. After removing 351 duplicate results, 1922 articles remained. Moreover, 1851 articles were excluded after reviewing the title and abstract, and 71 articles met the eligibility criteria for full-text review and further consideration. Finally, 26 of the 71 identified articles met the eligibility criteria. All selected studies were performed in Asian, American, and European countries (9 in China, 1 in India, 1 in Japan, 3 in the USA, 2 in South Korea, 1 in Belgium, 1 in Germany, 1 in Austria, 2 in Italy, 1 in Australia, 2 in the UK, 1 in Brazil, and 1 in Croatia). Platforms for proteomics included surface-enhanced laser desorption/ionization-time of flight mass spectrometry (SELDI-TOF-MS) (8 studies), SOMA scanning (1 study), electrospray ionization quadrupole time-of-flight mass spectrometry (ESI-Q-TOF-MS) (1 study), liquid chromatography‒mass spectrometry (LC‒MS/MS) (9 studies), and matrix-assisted laser desorption ionization-time of flight mass spectrometry (MALDI-TOF/MS) (7 studies). The biological samples included in this study were peripheral blood (15 studies), urine (8 studies), cervical mucus (1 study), and menstrual blood (2 studies). A PRISMA flow chart that depicts each step is shown in Fig. 1. The studies analysed in this review were all conducted from 2007 to 2023, and a total of 2,486 women were enrolled.

### Diagnostic accuracy of proteins

Various proteomic techniques have been used to investigate potential biomarkers for detecting endometriosis. Peripheral blood (serum and plasma) protein biomarker analysis has a sensitivity of 38–100% and a specificity of 59–99% for detecting endometriosis (Table 2). Additionally, urine proteomic profiling revealed that single and/or combined proteins could detect endometriosis with a sensitivity ranging from 58 to 91% and a specificity ranging from 76 to 93% (Table 2).

**Table 2:** Sensitivity and specificity of proteins/peptides detected from (A) peripheral blood (serum/plasma) and (B) urine in endometriosis

**A)**							
**Author (Year)**	Mw (kDa*) of proteins/peptides	**TP**	**FP**	**FN**	**TN**	**Sensitivity (95%CI)**	**Specificity (95%CI)**
Fassbender et al. 2012 a	2,831.02, 7,554.66, 4,241.29, 2,953.25, & 9,927.73	25	5	40	28	0.38 (0.27, 0.51)	0.85(0.68,0.95)
Fassbender et al. 2012 b	9,926.31, 10,072.2, 6,753.04, 4,302.67, & 9,328.49	18	4	27	18	0.4 (0.26, 0.56)	0.82(0.6,0.95)
Fassbender et al. 2012 c	11,365.3, 5,712.69, 10,070.7, 3,017.68 & 3,824.44	29	6	26	27	0.53 (0.39, 0.66)	0.82(0.65,0.93)
Seeber et al. 2010	1629.00 3047.00, 3526.00, 3774.00, 5046.00 & 5068.00	40	1	21	77	0.66 (0.52, 0.77)	0.99(0.93,1)
Wolfler et al. 2009	4159.00, 5264.00, 5603.00, 9861.00 & 10,533.00	40	16	11	23	0.78 (0.65, 0.89)	0.59(0.42,0.74)
Liu et al. 2009	3,956.00, 11,710.00 & 6,986.00	14	3	2	12	0.88 (0.62, 0.98)	0.8(0.52,0.80)
Liu et al. 2007	3,956.83, 11,710.70, & 6,986.45	32	4	4	28	0.89 (0.74, 0.97)	0.88(0.71,0.80)
Zhang et al. 2009	4974, 5813 & 4290	33	1	3	23	0.92 (0.78, 0.98)	0.96(0.79,1)
Zhang et al. 2013	4,180	29	3	0	32	1.00 (0.88, 1.00)	0.91(0.77,0.98)
**B**)							
**Author (Year)**	Mw (kDaa*)/name of proteins/peptides	**TP**	**FP**	**FN**	**TN**	**Sensitivity (95%CI)**	**Specificity (95%CI)**
Wang et al. 2014	1433.9, 1599.4, 2085.6, 6798.0 & 3217.2	10	1	1	14	0.91(0.59,1.00)	0.93(0.93,1.00)
Chen et al. 2019	Histone H4	18	14	7	58	0.72(0.51,0.88)	0.81(0.7,0.89)
Cho et al. 2012	VDBP-Cr	33	9	24	29	0.58(0.44,0.71)	0.76(0.60, 0.89)

*kDa; kilodalton, Mw; molecular weight

### Common DEPs in endometriosis

**Fig. 2:**
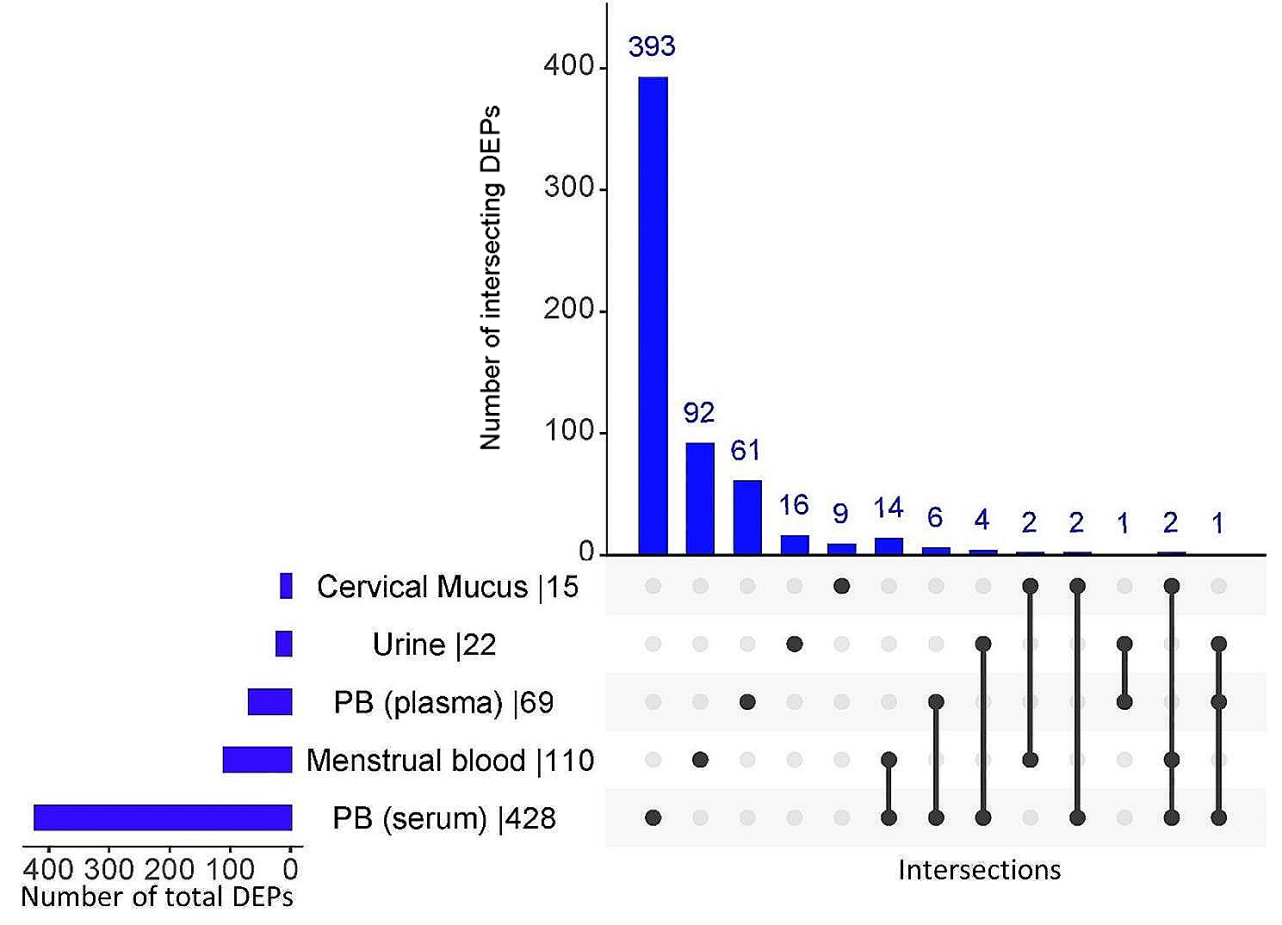
The distribution of DEPs (overexpressed) in endometriosis patients in different clinical samples supplemented with Table S1: List of differentially expressed proteins

In endometriosis, different proteins are expressed in various biological samples. Peroxiredoxin-6, angiopoietin-related protein, heterogeneous nuclear ribonucleoproteins, peroxiredoxin-1, leucine-rich alpha-2-glycoprotein, alpha-2-macroglobulin, apolipoprotein L1 and haptoglobin are commonly expressed proteins in plasma and serum samples. Alpha-1-antitrypsin, alpha-enolase, albumin, and vitamin D-binding protein are commonly expressed in both urine and serum, whereas S100-A8 and complement proteins are expressed in cervical mucus and menstrual blood as well as serum. Additionally, dynamin-1-like protein, rho GTPase-activating protein 6, rho GTPase-activating protein 18, zinc finger protein 185, FYN-binding protein 1, rho GTPase-activating protein 45, neurosecretory protein VGF, cartilage oligomeric matrix protein, stromal interaction molecule 1, polymeric immunoglobulin receptor, adipogenesis regulatory factor, complement C3, serum amyloid A-1 protein, fibrinogen gamma chain and ATP-dependent RNA helicase A are differentially expressed proteins in both serum and menstrual blood (Fig. 2 and Table S1).

### GO, KEGG, and PPI analyses of the DEPs in women with endometriosis

**Fig. 3:**
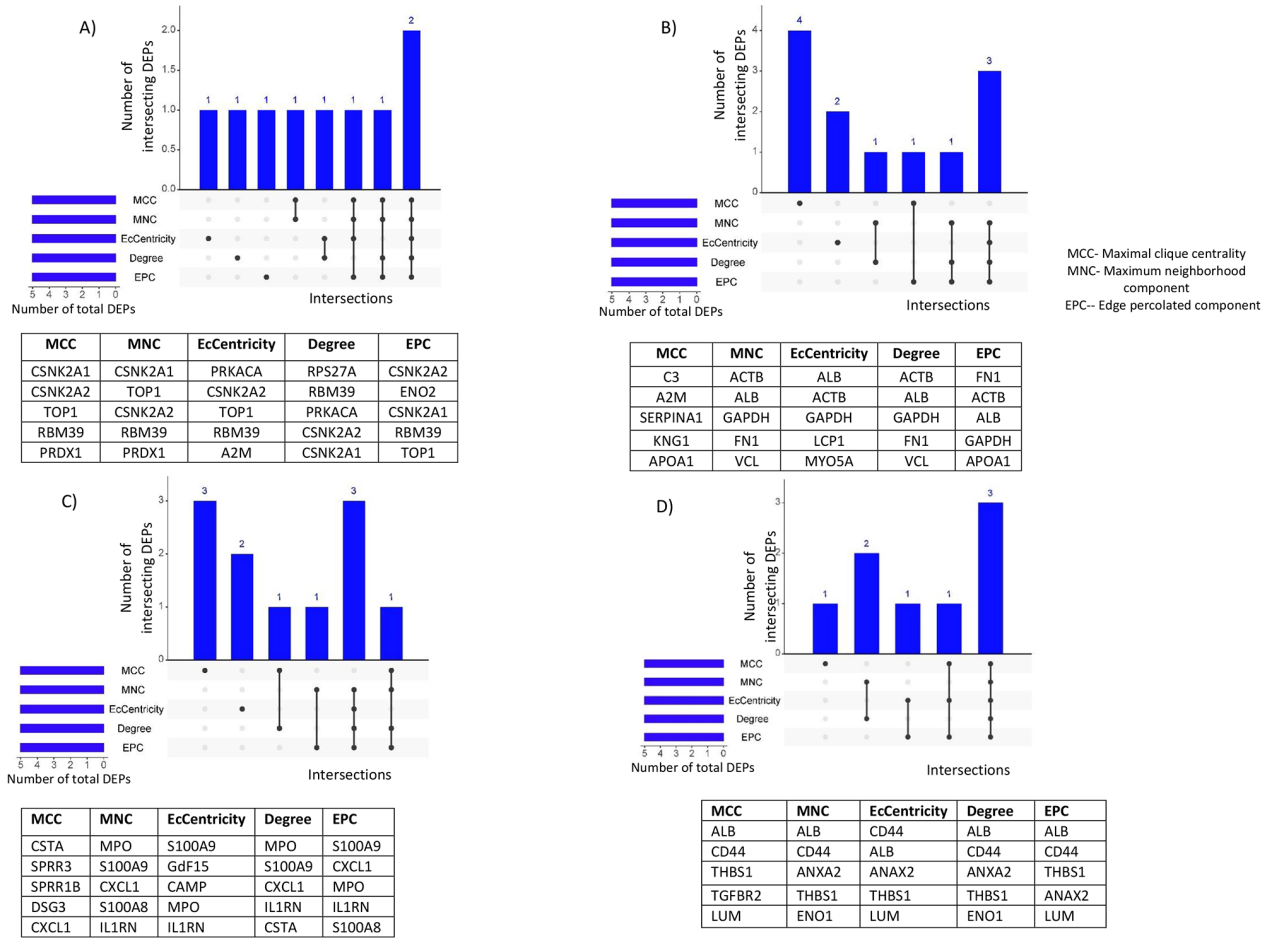
Top five DEPs in (**a**) plasma, (**b**) serum, (**c**) menstrual blood, and (**d**) urine of women with endometriosis

A total of 644 DEPs (180 upregulated and 464 downregulated) were identified from 9 studies in different clinical samples, such as peripheral blood (serum, plasma), menstrual blood, cervical mucus, and urine. Among these studies, 8 met the eligibility requirements for meta-analysis, and the remaining cervical mucus clinical samples were comprehensively reviewed and described (Fig. 3).

### Plasma

**Fig. 4:**
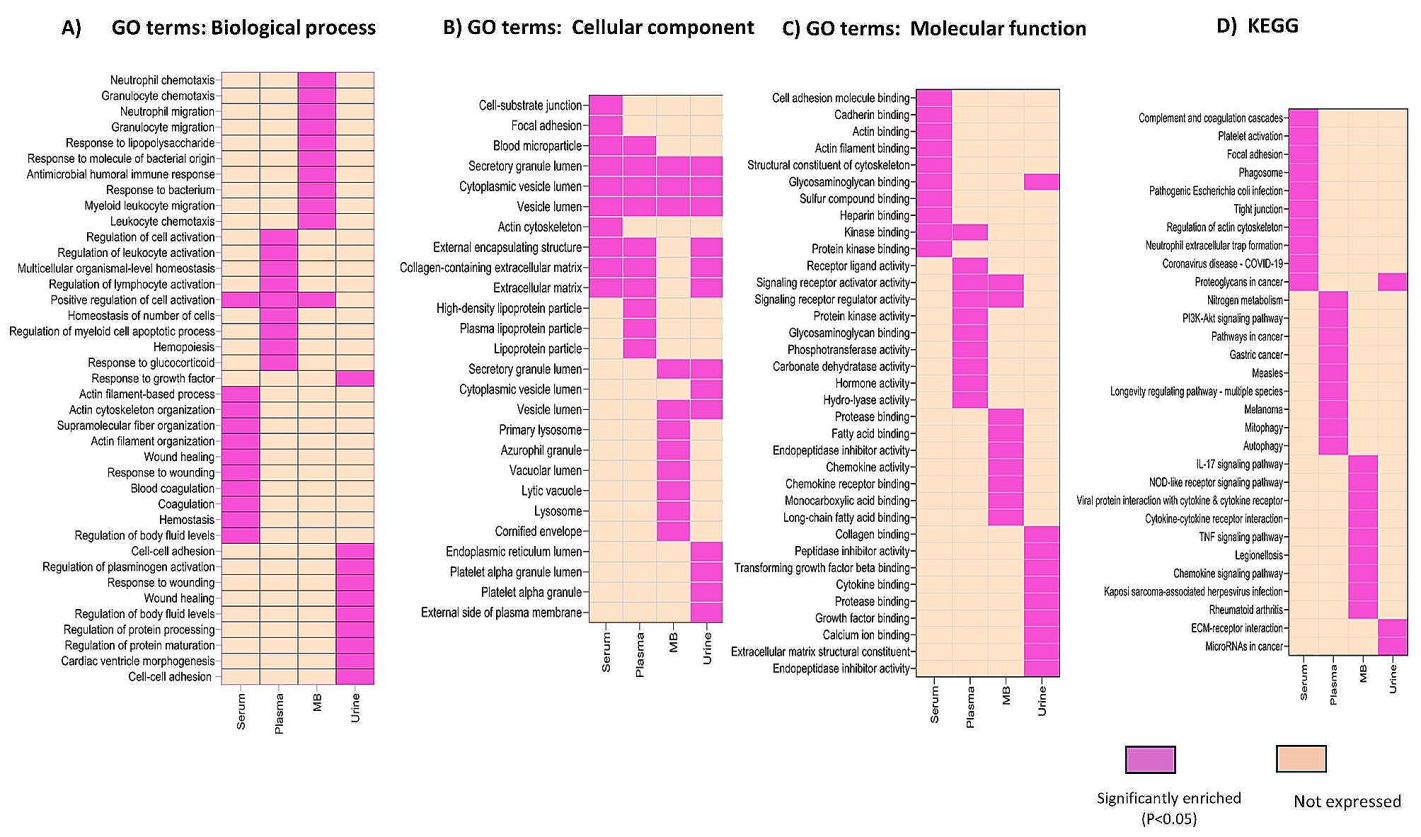
GO and KEGG analyses of the DEPs in women with endometriosis

The DEPs from plasma samples were analysed using GO terms that were categorized into molecular functions, cellular components, and biological processes. The molecular functions of the DEPs were primarily enriched in signalling receptor activator activity, signalling receptor regulator activity, and kinase activity. The GO terms in the cellular component category were mainly related to the collagen-containing extracellular matrix, the external secretory granule lumen, and the extracellular matrix. The biological process GO terms were primarily involved in the regulation of cell activation, regulation of leukocyte activation, and regulation of lymphocyte activation (Fig. 4 & Fig. S6). The enriched GO networks are also illustrated in Fig. S2.

We conducted KEGG pathway enrichment analysis of DEPs from plasma samples to explore DEP-related gene pathways in endometriosis. Nitrogen metabolism pathways, the phosphatidylinositol 3 kinase-protein kinase B (PI3K-Akt) pathway, and microRNAs in cancer pathways were the most significant (Fig. 4 & Fig. S7). In general, GO and KEGG analyses revealed that cell proliferation, adhesion, migration, and inflammation are involved in the pathophysiology of endometriosis.

PPI network analysis was performed for the 69 DEPs using the STRING database. After removing proteins without standard symbols, a total of 68 nodes and 121 edges were obtained that represented the interaction network with a *p* value of 1.98e-10. The top five hub genes identified using the cyto-Hubba plugin included casein kinase II subunit alpha (CSNK2A1, CSNK2A2), mammalian topoisomerase 1 (TOP1), cAMP-dependent protein kinase catalytic subunit alpha (PRKACA) and RNA-binding protein 39 (RBM39) (Fig. 2). The MCODE plugin distinguished two cluster networks, and all the top five hub genes, CSNK2A2, CSNK2A1, TOP1, PRKACA and RBM39, were included in the cluster with the highest score.

### Serum

Category-based GO analysis of the DEPs from serum samples was performed. The cellular component of the DEPs was predominantly enriched in collagen-containing extracellular matrix binding, extracellular matrix, and secretory vesicle lumen. The molecular function category was mainly involved in cell adhesion molecule binding, kinase binding, and actin binding (Fig. 4 & Fig S6). Actin filament organization, supramolecular fiber organization, and regulation of body fluid levels are predominantly involved in biological processes. The enriched GO term networks are also illustrated in Fig S3.

KEGG enrichment pathway analysis was carried out on serum samples to elucidate the pathogenesis of endometriosis. The top ten enriched pathways are illustrated in Fig. 4 and Fig. S7. The complement and coagulation cascades, platelet activation, neutrophil extracellular trap formation, and tight junction pathways were the most enriched KEGG pathways.

PPI network analysis of the 428 DEPs in serum was performed using the STRING database. A total of 396 nodes and 3186 edges associated with the PPI network were identified after removing proteins with no symbol name (PPI enrichment *p* value: < 1.0e-16). The five top hub genes were identified using the cytoHubba plugin and included albumin (ALB), actin, cytoplasmic 1 (ACTB), glyceraldehyde-3-phosphate dehydrogenase (GAPDH), fibronectin (FN1), and apolipoprotein A-I (APOA1) (Fig. 2). The MCODE plugin distinguished two cluster networks, and all the top five hub genes, ALB, ACTB, GAPDH, FN1, and APOA1, were included in the cluster with the highest score.

### Menstrual blood

GO analysis demonstrated that DEPs derived from menstrual blood are involved in the pathophysiology of endometriosis. In addition, the GO analysis results were categorized into three components, i.e., molecular functions, cellular components, and biological processes. The molecular functions of the DEPs were mainly enriched in protease binding, receptor‒ligand activity, and fatty acid binding. The GO terms in the cellular component category were mainly involved in the vesicle lumen, secretory granule lumen and cytoplasmic vesicle lumen (Fig. 4 & Fig. S6). Granulocyte migration, granulocyte chemotaxis, and leukocyte chemotaxis are the main biological processes involved. The enriched GO networks are also shown in Fig. S4.

The enriched KEGG pathways of DEPs from menstrual blood samples were used to further investigate DEP-related gene pathways. The top ten enriched pathways are illustrated in Fig. 4 and Fig. S7. IL-17 signalling pathway, complement and coagulation cascades, cytokine‒cytokine receptor interaction, and TNF signalling pathway. In conclusion, the GO and KEGG enrichment pathway analyses revealed that angiogenesis, cell proliferation, differentiation, and the induction of inflammation are highly important for the pathogenesis of endometriosis.

The STRING database was used for the PPI network analysis of 110 DEPs. After identifying proteins with no symbol name, there were 89 nodes and 134 edges associated with the PPI network (*p* value < 1.0e-16).

The top five hub genes identified using the cyto-Hubba plugin included protein S100 calcium-binding protein A9 (S100-A9), C-X-C motif chemokine ligand 1 (CXCL1), interleukin-1 receptor antagonist protein (IL1RN), cystatin-A (CSTA), and protein S100-A8 (Fig. 2). The MCODE plugin illustrated three cluster networks (cluster one: 7 nodes (desmoglein 1 & 3 (DSG1&DSG3), small proline-rich protein 3 (SPRR3), CSTA, small proline-rich protein 1B (SPRR1B), ajuba LIM protein (JUB) and serpin Family B Member 13 (SERPINB13), 19 edges; cluster two: 7 nodes (S100-A8, S100-A9, myeloperoxidase (MPO), IL1RN, C-X-C motif chemokine ligand 1 & 5 (CXCL1, CXCL5); and growth differentiation factor 15 (GDF15), 14 edges; and cluster three: 3 nodes (haptoglobin (HP), cyclic adenosine 3′,5′-monophosphate (CAMP) and resistin (RETN) and 3 edges). The top five hub genes, S100A9, IL1RN, CSTA, S100A8, and CXCL1, were included in the cluster with the highest score.

### Urine

The three categories of GO term analysis, i.e., molecular functions, cellular components, and biological processes, of the DEPs from urine samples were notably involved in the pathophysiology of endometriosis. The molecular functions of the DEPs were mainly enriched in collagen binding, cytokine binding, and transforming growth factor binding. The GO terms in the cellular components category were mainly involved in the collagen-containing extracellular matrix, secretory vesicle lumen, and extracellular matrix (Fig. 4 & Fig. S6). Cell‒cell adhesion, plasminogen activity regulation, and body fluid level regulation are the main biological processes involved. The enriched GO networks are also illustrated in Fig S5.

The KEGG pathway enrichment of DEPs from urine samples revealed the DEP-related gene pathways that are involved in the mechanism of endometriosis pathogenesis. ECM receptor interactions and microRNAs in cancer pathways were the pathways most significantly associated with endometriosis development (Fig. 4 & Fig. S7). Generally, GO and KEGG analyses revealed that cell growth and invasion, adhesion, and angiogenesis were implicated in the pathophysiology of endometriosis.

PPI network analysis of the 22 DEPs was performed using the STRING database, which revealed 22 nodes and 39 edges associated with the PPI network (*p* value: 9.7e-14). The top five hub genes identified using the cyto-Hubba plugin included thrombospondin-1 (THBS1), albumin (ALB), CD44 antigen (CD44), annexin A2 (ANXA2), and (LUM) (Fig. 2). The MCODE plugin distinguished two cluster networks. In cluster one, CD44, alkaline phosphatase (ALP), zinc-alpha-2-glycoprotein (AZGP1), alpha-1-antitrypsin (SERPINA1), ANAX2, and enolase 1 (ENO1) were the most sub connected proteins, whereas transforming growth factor beta receptor **2 (**TGFBR2), endoglin (ENG), THBS1 and LUM were the most highly connected subnetworks in cluster two.

## Discussion

This is a comprehensive systematic review and meta-analysis of proteomics data to explore common pathways and non-invasive diagnostic biomarkers for detecting endometriosis. Proteomic platforms offer an extraordinary opportunity to overcome the challenges associated with endometriosis by providing valuable insights into the mechanisms underlying the disease and identifying potential markers for diagnosis and therapeutic targeting. Hence, this study focused on recent improvements in proteomics technology aimed at identifying potential non-invasive diagnostic biomarkers and establishing mechanistic pathways to understand the pathogenesis of endometriosis.

### Alteration of proteins in endometriosis

This study investigated DEPs in peripheral blood, cervical mucus, menstrual blood, and urine from women with endometriosis. Although many proteins are altered in women with endometriosis, this review illustrates the common DEPs in diverse biological samples from women with endometriosis. DEPs commonly found in multiple biological samples, including vitamin D binding protein (VDBP), haptoglobin, S100-A8, cathepsin G, and complement component 3, are discussed.

VDBP is one of the most common proteins whose expression is altered in women with endometriosis. A line of evidence has shown that the expression of VDBP is substantially increased in the urine [37] and serum [38] of women with endometriosis compared to women without endometriosis. Similarly, the expression level of VDBP is markedly higher in endometrial tissue [39] but lower in peritoneal fluid [40] in women with endometriosis. Although studies have shown that VDBP may be implicated in the pathogenesis of endometriosis because of its chemotactic characteristics and ability to attract immune cells [39, 40], inconsistent patterns of VDBP expression have been observed across studies. The potential reasons for discrepancies may be observed in various studies, attributing them to differences in biological specimens, protein extraction procedures, centrifugal forces, and analysis platforms. Regarding the abundance of VDBP, studies have described diverse techniques for sample handling and analysis, such as 2DE-gel electrophoresis with LC‒MS/MS [37, 38] and ELISA [41, 42]. These disparities highlight the potential influence of methodological applications, as evidenced by (1) the superior sensitivity of LC‒MS/MS compared to ELISA, (2) the possibility of cell loss in the supernatant, affecting the abundance and concentration of proteins when employing low centrifugation force or short processing time, and (3) the superior sensitivity and ability of ELISA to detect very small amounts of target proteins compared to 2DE-gel electrophoresis [43, 44]. These perspectives highlight the clinical utility of LC‒MS/MS, which is a standard and high-throughput proteomics technology with a lesser tendency for bias or interference, as well as greater quantitative agreement among laboratories and biological samples [45, 46]. Given the wide range of variation within biological samples that does not adequately explore protein alterations across the severity and phenotype of endometriosis, conducting further large-scale multi-omics studies would be helpful to elucidate the association between VDBP and the underlying mechanism of endometriosis.

The expression level of haptoglobin decreased in the plasma and serum of women with endometriosis [47]. However, this finding contradicts the findings of Wölfler et al., who demonstrated that the alteration of haptoglobin is significantly increased in the peritoneal fluid of patients with ovarian and peritoneal endometriosis [48]. The potential variation may be due to the diverse phenotypes of endometriosis, including ovarian, peritoneal, and deep endometriotic lesions, as well as the timing of sample collection. The upregulation of estrogen and the estrogen receptor on macrophages in the peritoneal cavity generates an abnormal immune microenvironment, potentially resulting in increased haptoglobin production [49]. In addition to the phenotype of endometriosis, the depletion of proteins should also be considered for the variations that ensue. Some studies depleted the most abundant proteins, such as albumin and globulin, to detect low-abundance proteins, which may be putative disease biomarkers in biological samples [47], whereas other studies did not mention the depletion process during protein extraction and identification [50–52]. Therefore, protein depletion can affect the haptoglobin concentration during protein extraction via different mechanisms, including reduced solubility, altered protein‒ligand interactions, and competitive binding [53–55]. The proteomics analysis platform is also another confounding factor. The two common analysis platforms are mass spectrometry and enzyme-linked immunoassay. Both techniques are used to detect the concentration and expression of proteins. However, compared with ELISA, mass spectrometry (MS)-based proteomics analysis [47, 50] provides high accuracy, resolution, reproducibility, and sensitivity in identifying and quantifying proteins in a complex mixture, often not allowing differentiation between the peptide and its derivatives or degradation fragments [49, 56].

This study similarly demonstrated that the protein S100-A8 is markedly reduced in the cervical mucus of women with endometriosis. This finding supports the findings of a study conducted in France, which identified S100-A8 as a promising endometrial diagnostic marker for both the proliferative and secretory phases [57]. Additionally, another study showed that S100A8 is predominant in the peritoneal fluid of women with early-stage deep endometriosis [51]. In addition, the presence of higher levels of S100A8 in the peritoneal fluid of women with endometriosis suggests its potential contribution to the development and formation of lesions within the peritoneal cavity through inflammatory pathways by activating neutrophils [58, 59].

This study also revealed that cathepsin G is a common DEP in the urine, serum, and plasma of women with endometriosis. This finding supports the findings of a study conducted in Poland, which revealed that cathepsin G is significantly elevated in the endometrial tissue of women with endometriosis and may play a role in disease development and progression [60]. Several lines of evidence have demonstrated that cathepsin G plays an essential role in the pathogenesis of endometriosis by promoting extracellular matrix degradation and invasion [61], activating collagen production [61], and stimulating the inflammatory process [62], which facilitates the implantation and growth of endometrial tissue outside the uterus.

This comprehensive study also showed that complement C3 levels are significantly higher in women with endometriosis than in those without endometriosis. Similarly, it has been reported that the abundance of C3 is significantly higher in peritoneal fluid [63] and endometrial tissue [64, 65] in women with endometriosis. The involvement of complement C3, as expressed by ectopic endometrial tissue, in the formation of endometriotic lesions is mediated by mast cell activation. Additionally, it may be generated locally by ectopic endometrial tissue and can promote the engraftment of endometriotic cysts [65, 66]. Moreover, cyto-hub gene analysis revealed that CSNK2A1, CSNK2A2, TOP1, PRKACA, RBM39 (plasma), ALB, ACTB, GAPDH, FN1, APOA1 (serum), S100-A9, CXCL1, IL1RN, CSTA, S100-A8 (menstrual blood) and THBS1, ALB, CD44, ANXA2, and LUM (urine) were the top 5 proteins expressed in women with endometriosis. Among all the proteins, ALB is commonly expressed in both serum and urine. These disparities were also revealed by a study conducted by Donal S et al., who reported that the percentages of proteins in venous blood, menstrual blood, and vaginal fluid were 61%, 36%, and 35%, respectively. These body fluid-derived proteins could contribute to augmenting the diagnosis of endometriosis combined with imaging techniques and physical examinations. Nevertheless, to enhance the diagnostic accuracy of non-invasive biological sample-derived proteins, further comprehensive functional and validation multi-omics studies with large sample sizes are needed.

GO analysis revealed that the modulation of molecular, functional, and cellular processes contributes to the pathophysiology of endometriosis through the activation of the collagen-containing extracellular matrix, extracellular matrix, secretory granule lumen, and others [67]. These GO terms play a role in cell migration, adhesion, angiogenesis, immune response, lymphocyte activation, tissue survival, and facilitating the implantation and potential growth of ectopic endometrial lesions [13, 68–71].

KEGG enrichment analysis revealed that nitrogen metabolism [72], PI3K-Akt [73], platelet activation [74], the NOD-like receptor signalling pathway [75], ECM-receptor interactions [76], cytokine‒cytokine receptor interactions [76], IL-17 signalling [77], complement and coagulation cascades [78], TNF signalling and proteoglycans in cancer [79] have been implicated in the pathogenesis of endometriosis. These pathways play a significant role in the cellular growth and survival of endometriotic lesions [80–82]. The ECM pathway plays a key role in cell migration, adhesion, and tissue remodelling through the modulation of matrix metalloproteinases that interact with various growth factors and proinflammatory cytokines, such as transforming growth factor-beta (TGF-β), interleukin-1 (IL-1), and tumor necrosis factor-alpha (TNF-α) [83–85].

The NOD-like receptor pathway is an important signalling pathway that is involved in the pathogenesis of endometriosis [86]. This pathway encompasses the family pyrin domain containing 3 (NLRP3), an intracellular receptor that initiates the release of proinflammatory cytokines such as interleukin-1β (IL-1β) upon the activation of NLRP3. Abnormal activation of the NLRP3 inflammasome has been observed within ectopic endometrial lesions, peritoneal fluid, and the eutopic endometrium of women with endometriosis. This dysregulated activation significantly contributes to persistent inflammation and accompanying pain related to the condition [86–89]. Cytokine‒cytokine receptor interactions and the IL-17 signalling pathway have been implicated in the pathogenesis of endometriosis. IL-17 has been shown to promote the production of other proinflammatory cytokines, such as IL-1α and IL-1β, involved in the pathogenesis of endometriosis [77]. Additionally, an interaction between the complement system and coagulation system might contribute to the pathophysiology of endometriosis following the monthly shedding of endometrial tissues, triggering complement activation resulting from the activation of the microenvironment in women diagnosed with endometriosis [90].

Proteoglycans involved in cancer pathways are commonly enriched in both the serum and urine of women with endometriosis. Proteoglycans are complex molecules that are secreted by cancer cells and stromal cells and are composed of glycosaminoglycan (GAG) chains [91]. The literature has shown that proteoglycans play a significant role in regulating cell-to-cell and cell-to-matrix interactions, releasing growth factors and cytokines that can promote cell proliferation and invasion [92]. Hence, the trapping and release of angiogenic factors and cytokines that trigger proliferation and invasion are implicated in the pathophysiology of endometriosis.

**Fig. 5:**
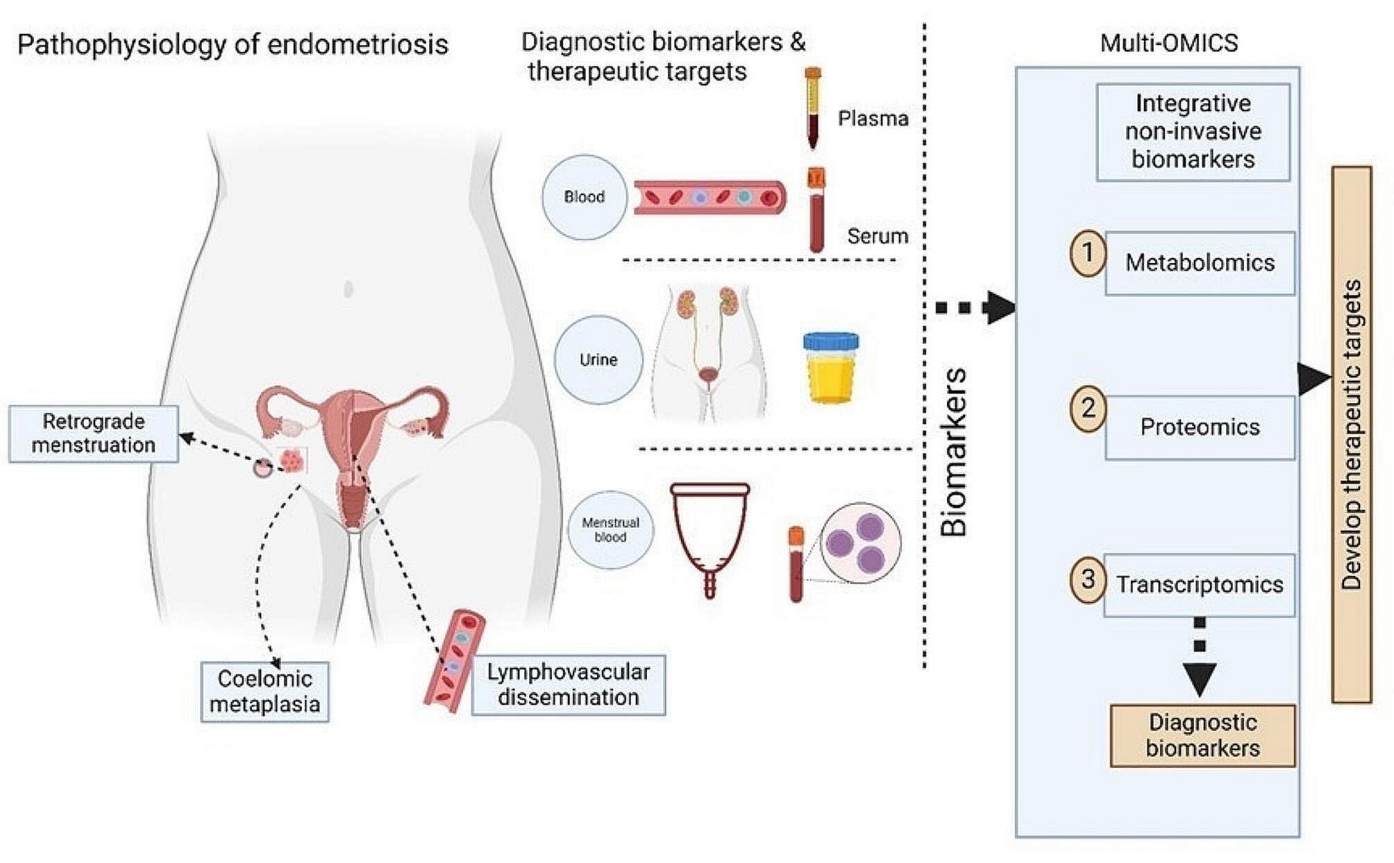
Newly proposed approach for the integrative study of endometriosis

Overall, this proteomics study provides insights into the expression of common and distinct proteins that are expressed in women with endometriosis. Given the different conditions of the study participants, the phenotype and severity of endometriosis, sample handling, and processing methods, proteomic platforms, and different menstrual cycles, we recommend the use of an integrated multi-OMICS study in which all non-invasive biological samples from the same patients are adjusted for confounders to enhance the mechanism of disease development and provide an opportunity to identify novel diagnostic and therapeutic targets for endometriosis (Fig. 5).

### Strengths and limitations

This is a comprehensive systematic review and meta-analysis to explore the applicability of the proteomics approach to discover novel diagnostic biomarkers and unravel therapeutic targets from non-invasive biological samples. Additionally, this study serves as an input for further multi-OMICS studies to uncover and establish novel diagnostic and therapeutic targets in endometriosis. There are some limitations in our study. First, there is a lack of sufficient studies on the overall diagnostic accuracy of individual or combined proteins based on the expression molecular weight of proteins/peptides in different phases of the menstrual cycle. Although the literature has shown protein expression in endometriosis during different phases of the human menstrual cycle, the difference in protein expression between the proliferative and secretory phases remains controversial. Therefore, further evidence is required to explore the diagnostic accuracy of protein biomarkers concerning the m/z ratio in different phases of the menstrual cycle. Second, the lack of available raw data and/or full protein lists allowed us to focus only on the differentially expressed protein lists, which could affect the conclusions of the findings. Additionally, the lack of studies did not allow us to look at the differentially expressed proteins across the stages (early vs. advanced, subtypes of endometriosis (ovarian, peritoneal & deep infiltrating) and menstrual cycles (secretary, proliferative and menstrual phases).

## Conclusion

In summary, this comprehensive meta-analysis of differentially expressed proteins from non-invasive clinical samples highlights the pathophysiology of endometriosis with GO and enriched KEGG pathways. Moreover, proteomics holds promise for the discovery of peripheral blood, menstrual blood, cervical mucus, and urine-based biomarkers for endometriosis. Various upregulated and downregulated proteins have been identified, suggesting their potential utility as promising non-invasive biomarkers for endometriosis detection and disease development mechanisms.

Furthermore, this review explored how the expression of different proteins and pathways in multiple clinical samples from non-invasive sources can be used to elucidate the pathophysiology of endometriosis. Finally, our findings provide new knowledge that will be helpful in understanding the pathophysiology of endometriosis, and future integrated studies involving peripheral blood, menstrual blood, and urine samples are needed. The identified proteins and pathways not only expand our understanding of the disease but also offer promising targets for future research. Furthermore, validation of these findings, exploration of hub genes for diagnostic accuracy, and further research across a wider range of samples and endometriosis types are key to revealing new options for non-invasive diagnosis and helping to explore more effective potential treatment options. Moreover, further research is needed to validate these findings and potentially help to improve the diagnosis, enhance pathophysiology, and offer hints for potential treatments for endometriosis.

### Electronic supplementary material

Below is the link to the electronic supplementary material.

**Supplementary Material 1**: Figure S1. QUADAS-2 tool: The distribution of risk-of-bias (A) and applicability (B) judgments within each bias domain. Figure S2. Network of enriched GO terms in peripheral blood (plasma): (a) biological process, (b) cellular component and (c) molecular function. Figure S3. Network of enriched GO terms in peripheral blood (serum): (a) biological process, (b) cellular component and (c) molecular function. Figure S4. Network of enriched GO terms in menstrual blood. (a) biological process (b) cellular component and (c) molecular function. Figure S5. Network of enriched GO terms in urine: (a) biological process, (b) cellular component and (c) molecular function. Figure S6. GO term analysis of DEPs in plasma, serum, menstrual blood, and urine from patients with endometriosis

**Supplementary Material 2**: Table S1. List of differentially expressed proteins

## Data Availability

The data underlying this article are available upon the request of the corresponding authors.
