# Supplementary material for: Proteomics approach to discovering non-invasive diagnostic biomarkers and understanding the pathogenesis of endometriosis: a systematic review and meta-analysis
Source: J Transl Med. 2024 Jul 26;22:685. doi: 10.1186/s12967-024-05474-3 (PMC11282838; doi:10.1186/s12967-024-05474-3)
Supplement: Supplementary file 1 — Supplementary Material 1: Figure S1. QUADAS-2 tool: The distribution of risk-of-bias (A) and applicability (B) judgments within each bias domain. Figure S2. Network of enriched GO terms in peripheral blood (plasma): (a) biological process, (b) cellular component and (c) molecular function. Figure S3. Network of enriched GO terms in peripheral blood (serum): (a) biological process, (b) cellular component and (c) molecular function. Figure S4. Network of enriched GO terms in menstrual blood. (a) biological process (b) cellular component and (c) molecular function. Figure S5. Network of enriched GO terms in urine: (a) biological process, (b) cellular component and (c) molecular function. Figure S6. GO term analysis of DEPs in plasma, serum, menstrual blood, and urine from patients with endometriosis [file 12967_2024_5474_MOESM1_ESM.docx]

1. B)

REFERENCE STANDARED

INDEX TEST

PATIENT SELECTION

**Figure S1**

**Intersections**


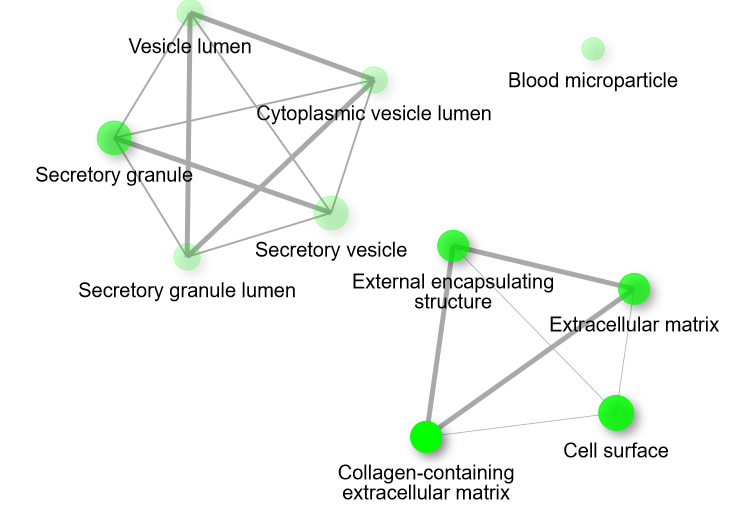

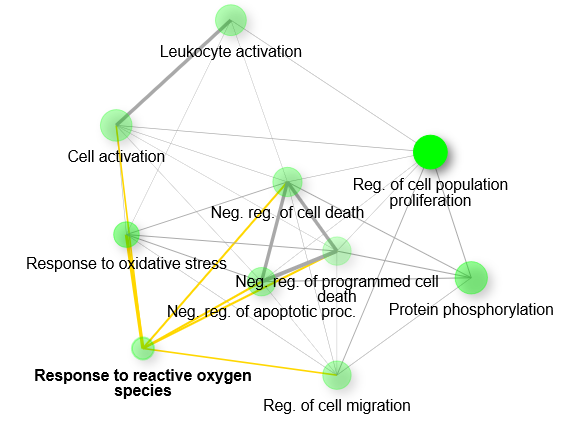
A) B)

C C)


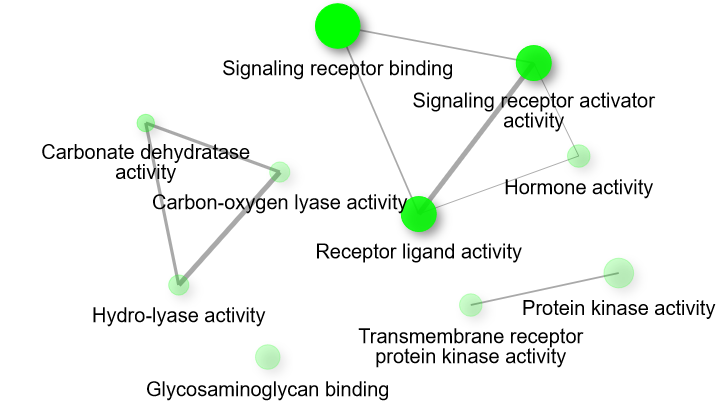


Darker nodes show more significantly enriched gene sets, bigger nodes represent larger gene sets and thicker edges represent more overlapped genes. **Figure S2**


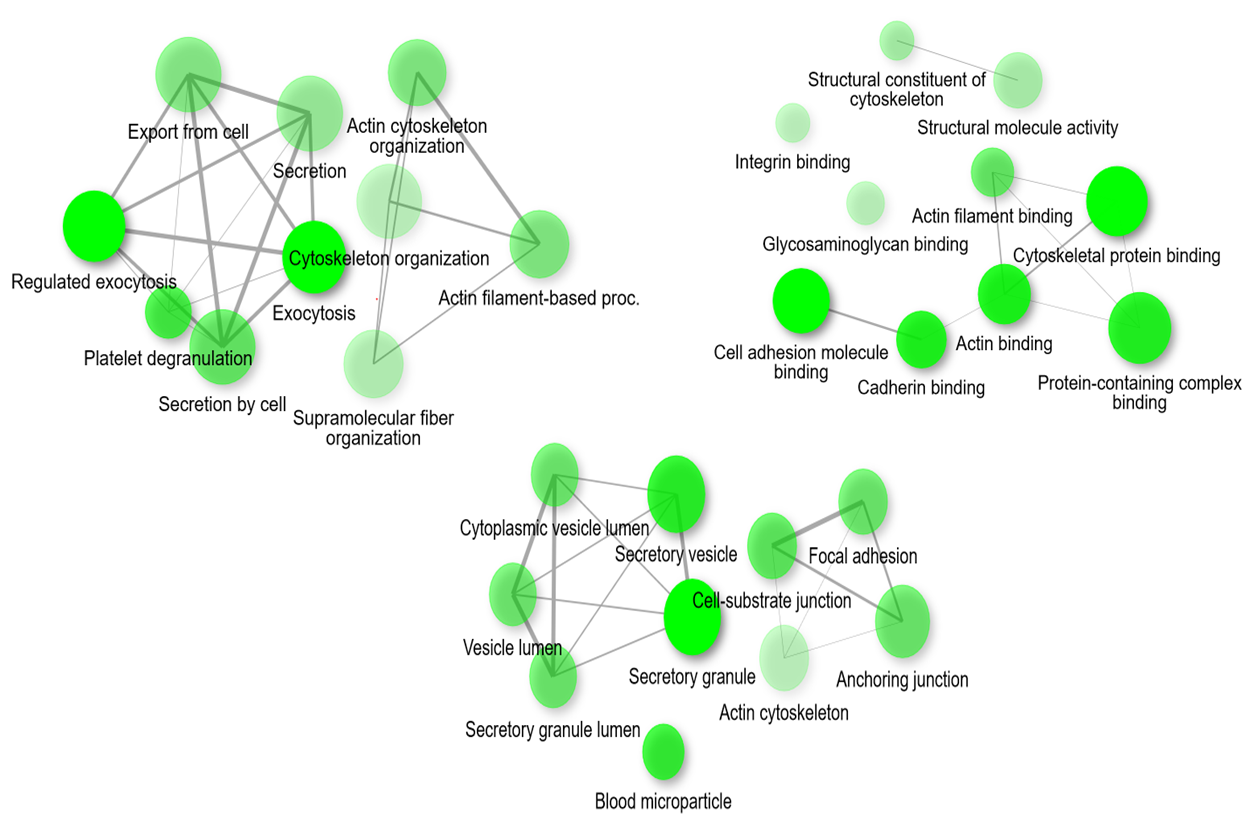


B)

A)

C)

Darker nodes show more significantly enriched gene sets, bigger nodes represent larger gene sets and thicker edges represent more overlapped genes.

**Figure S3**


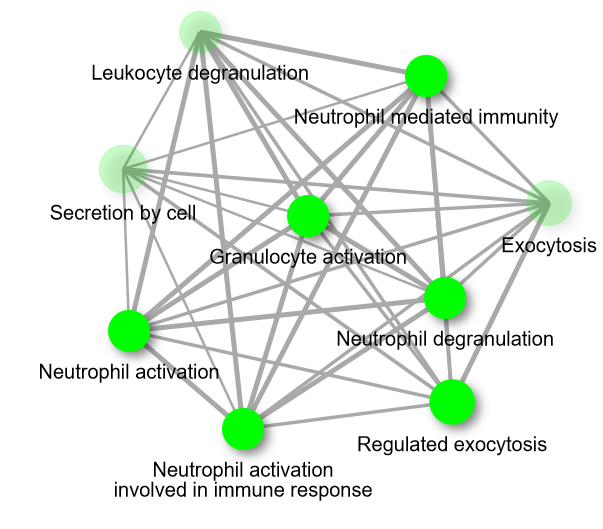

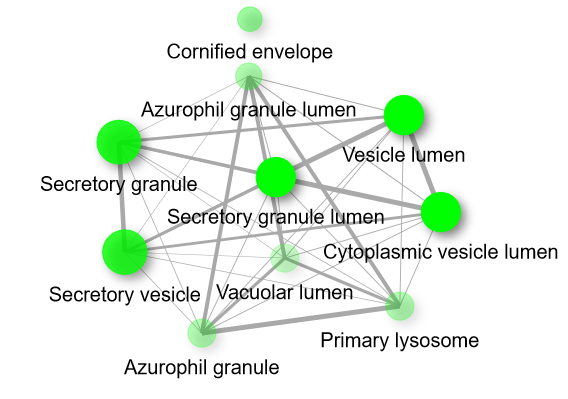


B)

A)

C)


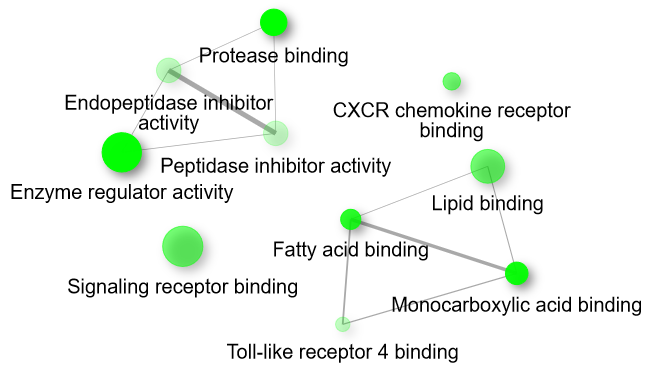


Darker nodes show more significantly enriched gene sets, bigger nodes represent larger gene sets and thicker edges represent more overlapped genes).

**Figure S4**


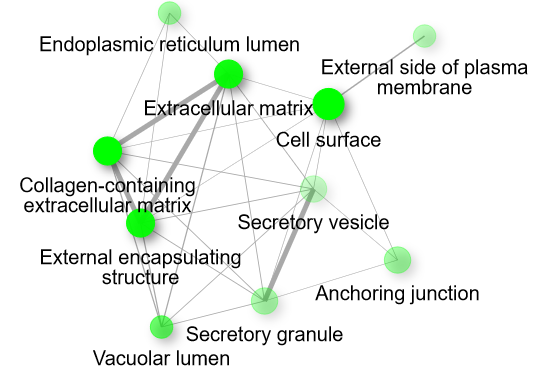

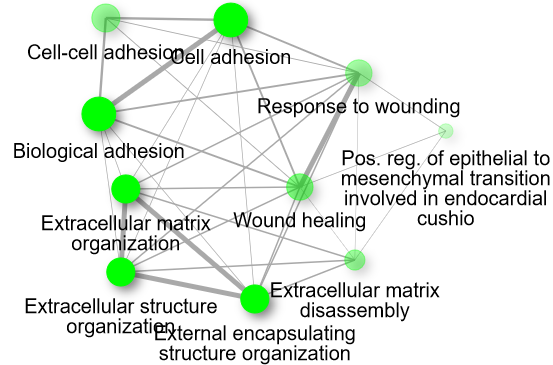


B)

A)

C)


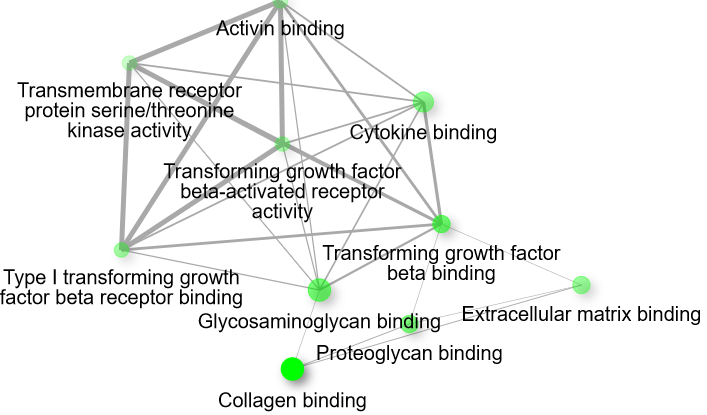


BP—Biological process

CC—Cellular component

MF—Molecular function

Darker nodes show more significantly enriched gene sets, bigger nodes represent larger gene sets and thicker edges represent more overlapped genes

**Figure S5**


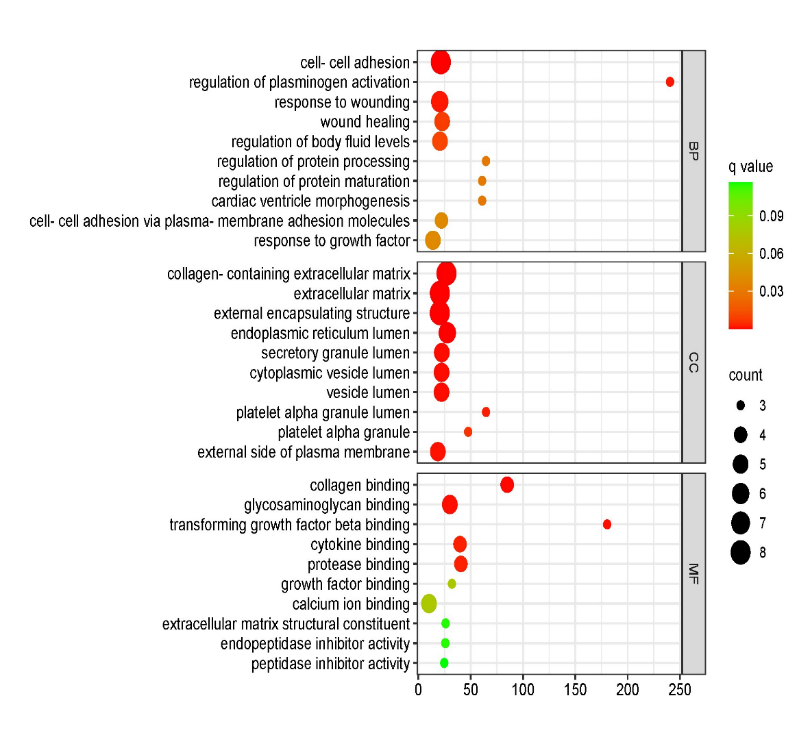

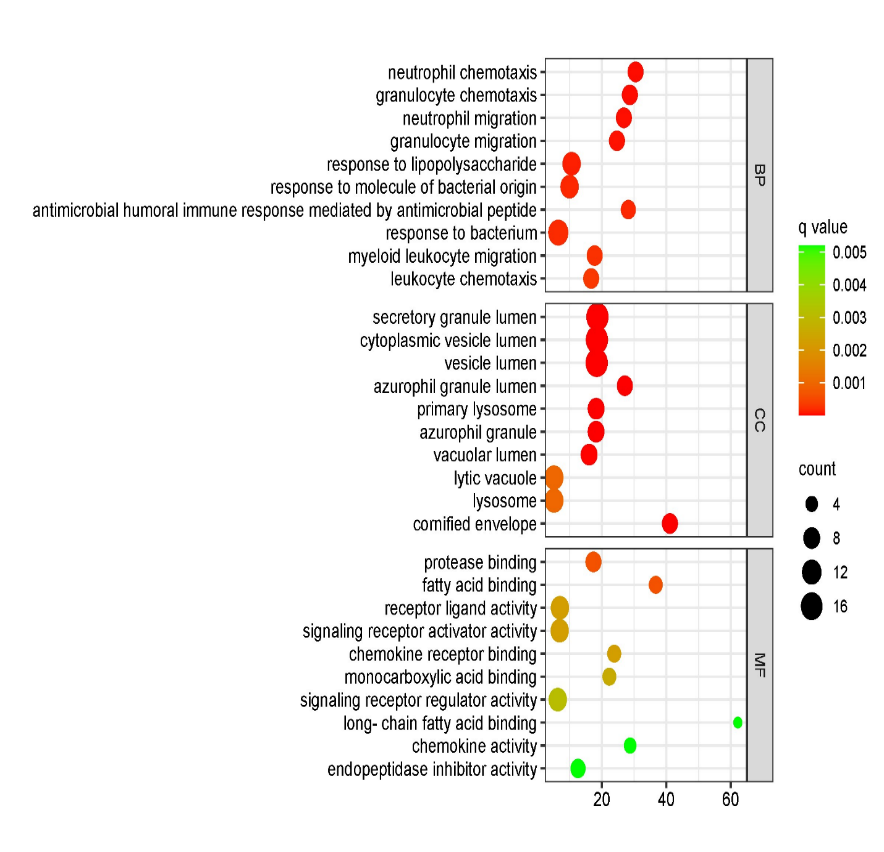

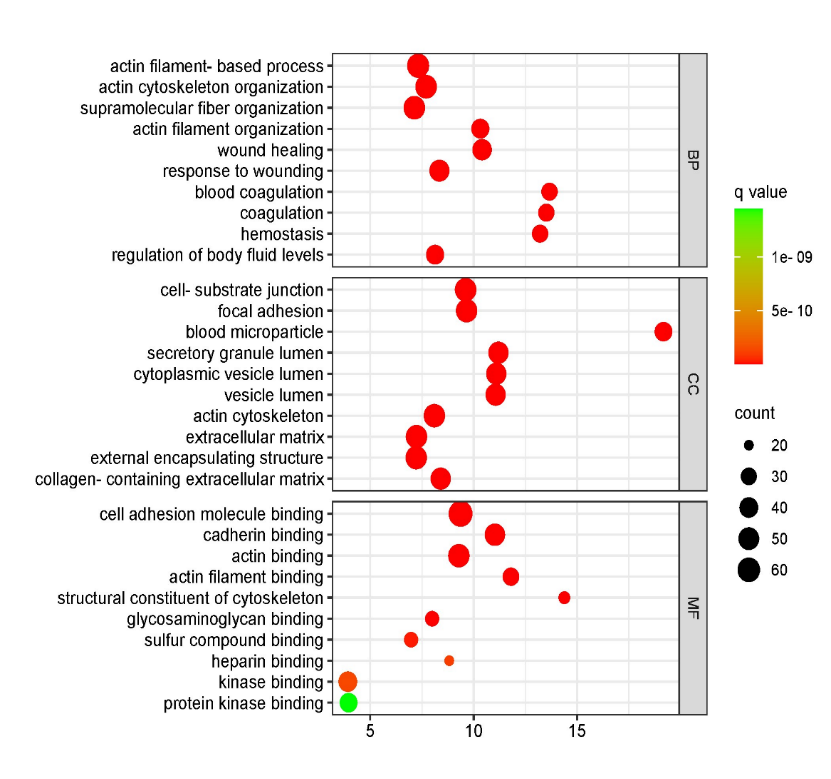

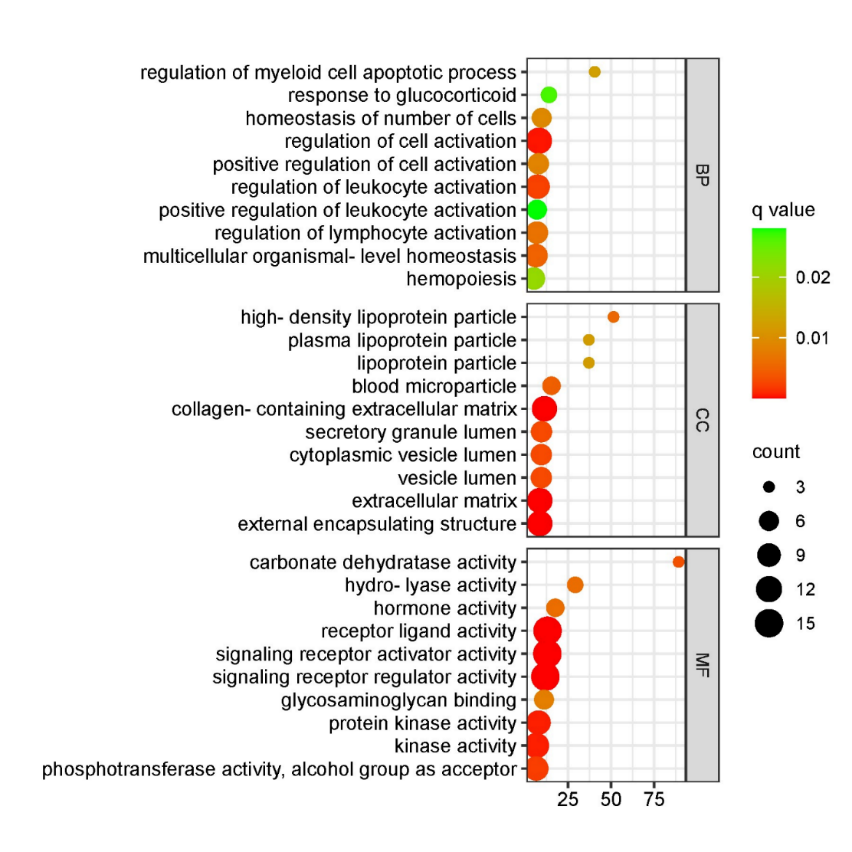


D)

C)

**Fig S6**

B)

A)


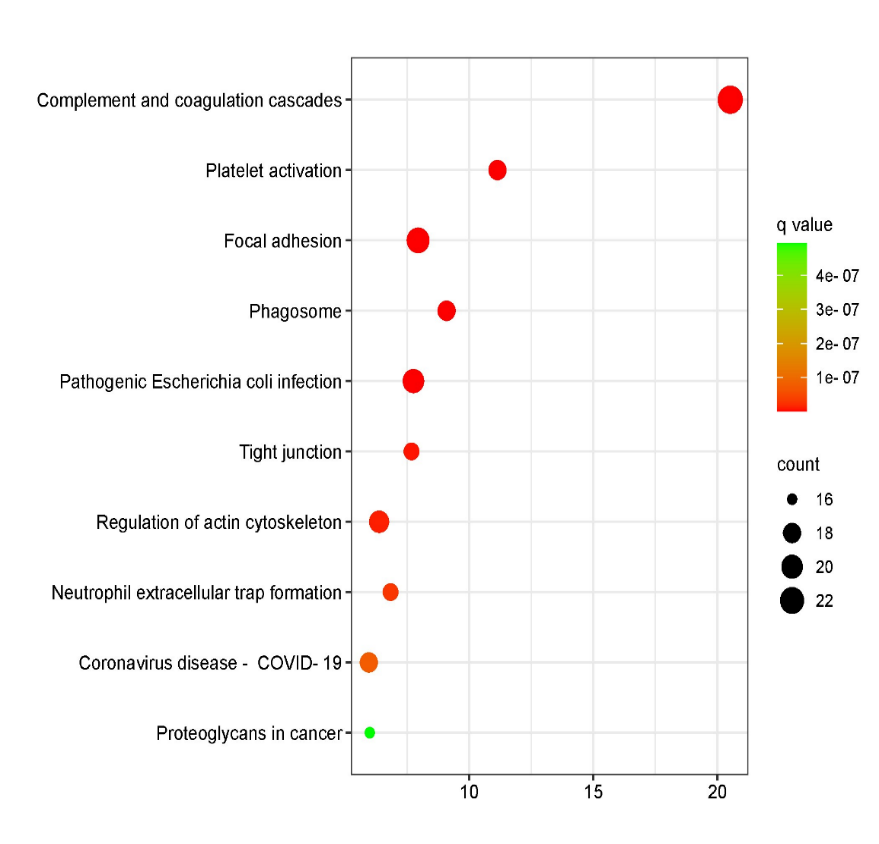

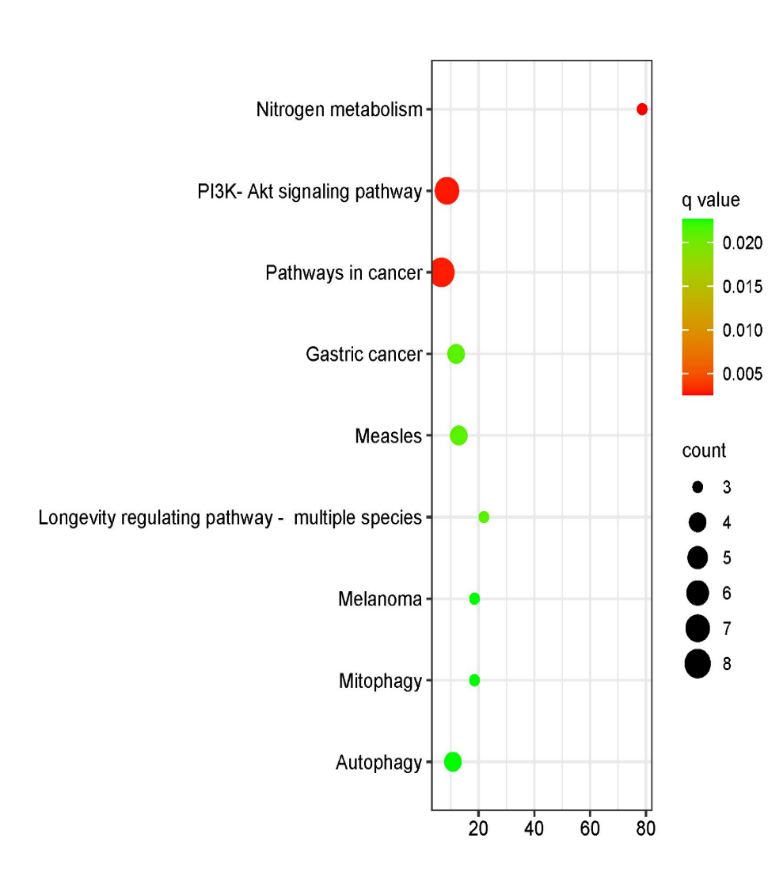

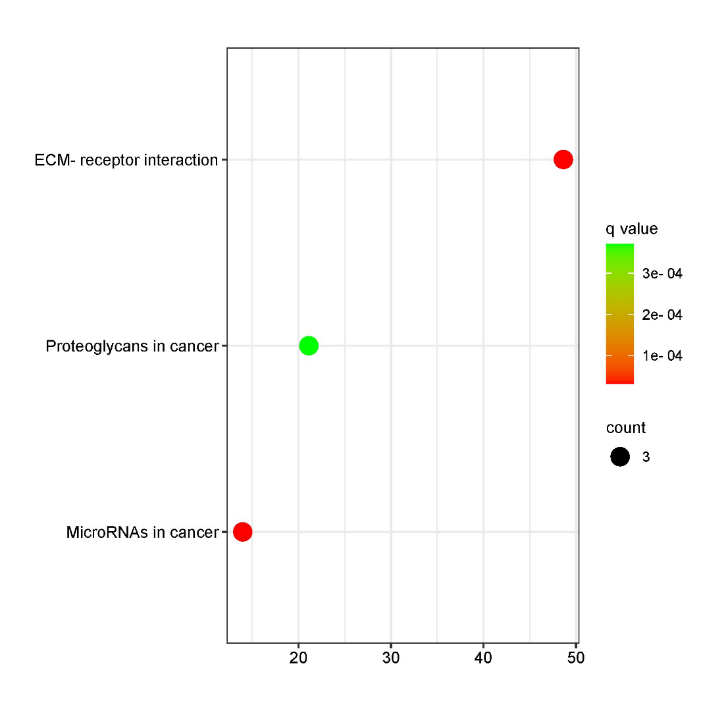

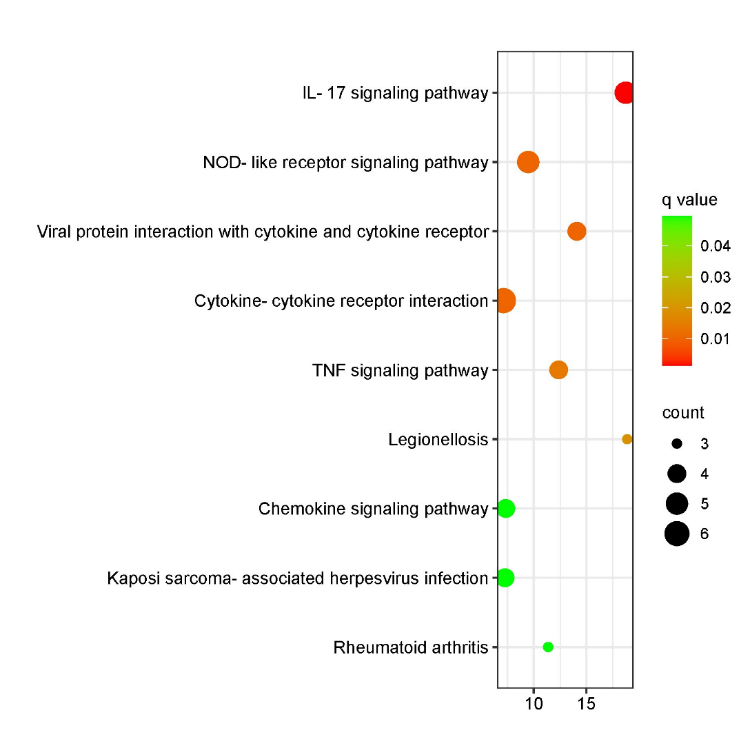


D)

C)

**Fig S7**
