# Supplementary material for: Proteomics approach to discovering non-invasive diagnostic biomarkers and understanding the pathogenesis of endometriosis: a systematic review and meta-analysis
Source: J Transl Med. 2024 Jul 26;22:685. doi: 10.1186/s12967-024-05474-3 (PMC11282838; doi:10.1186/s12967-024-05474-3)
Supplement: Supplementary file 2 — Supplementary Material 2: Table S1. List of differentially expressed proteins [file 12967_2024_5474_MOESM2_ESM.docx]

**Table S1**

| Biological samples | Protein name (Uniport ID) |
| --- | --- |
| Serum | P61218, Q9H939, P25685, P00709, Q27J81, Q9UJC5, P61006, Q9NR12, P06753, P0DMM9, Q05209, Q9ULV4, O60673, Q15555, P50502, O00429, P58546, Q9UBC2, Q9H4B7, Q99426, P48444, P01614, Q008S8, Q9NY65, P46109, P13196, P18206, P54727, P41439, Q14696, Q9NRW1, Q14677, Q9NZN3, Q9P1F3, O43182, Q5SQ64, Q14247, Q9NYL9, Q9BR76, Q15691, O75563, Q0VAK6, O00151, Q9P0Z9, P26641, Q05682, Q99439, Q9ULU8, P67936, Q0ZGT2, Q15149, O95747, P52566, Q8NFY9, Q16643, Q9H4M9, Q9UBW5, Q9H299, P50552, Q86UX7, Q3ZCW2, O75445, O43639, P27169, Q9Y251, Q9HBI1, P31644, O00194. Q9BT92, Q9C0C9, P37802, O00161, Q9Y490, P61224, P11047, P68366, O95866, P09493, Q6ZU35, P62328, O14960, Q8N392, Q9UEY8, Q9HCK1, Q5M775, Q08ET2, Q6KC79, P55145, P31151, Q14019, Q13813, O15126, P35579, Q15365, O75822, Q4KMP7, O15231, Q7RTZ1, O15117, Q14847,Q9UDY2, Q5JSH3, P82094, Q9NQ75, P50402, P30044, P07437, Q9Y371, Q9P0L0, P62993, P07737, Q86YW5, P21333, O60496Q96SN8, P21291, O75558, A6NJ16, Q15166, P16284, P98172, P11226, P51149, O75791, O95445, O14745, Q9Y5K6, Q7LDG7, Q92619, Q6ZVM7, Q13442, P23588, Q9UJU6, Q01518, O95810, O43294,P61106, P26038.P12318,P19957, Q14203, P02647, Q969T9, P35908, Q9NPH3, Q15942, P49908, P31431, P60660, P41229, P04632, Q9UHB6, P04792, Q9UNW1, Q9NZM3, P23280, Q9Y4I1, O95721, Q16661, O14974, P52565, P51452, P35611, P14317, Q8ND07, Q6IBS0, P11684, P62937, Q9UNZ2, Q01082, O60645, O95989, O43516, Q14574, Q9NZJ4, P59998, O76074, O75015, P12081, Q15848, P08571, P08709, O43399, Q14008, P26885, P35443, P68036, Q12794, P01702, P07108, P04196, P50281, P01607, P03952, P01042, P20340, P23528, P02774, P10644, Q96AA8, P05160, Q96BY9, Q15843, P23284, P07359, P13693, P55196, P49767, O95047, Q7Z3B1, P10599, Q13555, P14151, P42566, P26927, P05090, Q15147, P60709, Q9UIB8, Q16760, P25774, P02654, P07195, Q96S96, P98196, O15145, P07148, P60985, Q96L73, O00187, Q9Y5C1, Q14520, P27918, P04156, P05556, P10643, P02671, Q14118, P29279, P28300, Q96JA1, Q8N4N8, P19823, P01860, Q9BRK3, P02746, A1L4H1, P04004, P10124, Q9ULI3, P58166, Q04721, O15240, P02788, P01606, P13727, O00391, P01707, Q13232, Q9Y6R7, P05787, P06318, P06703, P01602, Q9BXT8, P01604, P06576, P01610, O00534, P49747,P30049, P43121, O76090, P01598, P01714, P01705, P07307, P02545, O75094, Q13586, Q08397, P04406, P01833, Q96NZ9, Q9H1Z8, P18850, Q15847, P06733, P04209, P01024, P46782, P01903, P30041, P01710, O60234, Q8IZA0, P00167, P41159, P01712, P43251, P04208, P20700, P02675, Q6GTX8, Q4G0P3, P49006, P0DJI8, Q9NYF8, P00441, P02679, Q9Y279, Q8IUL8, P30046, O43915, P28827, Q9BXX0, P30043, P18428, P05387, Q9BUN1, P23083, P13796, Q9Y2V2, Q04760, Q06830, P15311, P02751, Q8IZT6, P01715, Q9BYT8, P02741, P01708, P00738, P29622, P01023, Q8WXH0, P32119, P68871, P51693, P25705, P23142, Q9P2R6, P08670, P02786, P61604, O14782, P02748, Q96JI7, P06748,P05114, P16949, P01861, Q9P0N9, P07911, O15031, Q8NF91, P01160, P01871, O95153, P09651, P51991,P84103,Q3YBR2, P26373, P16403, O43423, P08311, P33908, Q13151, P01591, P04275, O43866, P62263, Q9NZR2, P22626, P0C0L4, Q08211, Q01433, Q01105, P51161, P16401, P09429, P08294, Q9BYP7, Q8N2N9, P62805, P16402, O60814, P19338, P42167, P06899, Q92522, P01011, P07910, P05154, Q5VT06, Q9P2D3, P16104, Q8WUW1, O94915, P05106, O00443, A2RUB6, P59923, P43652, P01011, Q14624, P00751, P01834, P00738, Q9C035, P02768, P02790, O95445, P01876, O14791, P04217, P02749, P02743, P02787, P01009, P01023, P08603, P02748, P14136, P10909, P02647, P02750 |
| Plasma | P01275, Q9NZ08, Q9H773, P51654, P17612, Q9HBG7, Q6YHK3, P02794, P17813, Q9H3T3, P81172, P09104, P17181, P30041, Q15768, Q9Y5C1, Q14498, O60565, P14210, P08311, Q29983, O14836, P22304, P04083, P04278, P01588, P43490, P11171 , Q9Y4C5 , P41217, P10415, Q13131, Q08431, P04040 , Q9NQW7, P00568, P62979 , P21246 , P01298, P22626, P55075, Q99706, P11387, P10915, Q99729, Q8N1Q1, P01854, P12034, Q9BXN2, P42679, P19784, P51813, P56470, P14780, Q16832, P28325, P07451, P10082, Q06830, P00915, P68400, P20226, P58499, P02649, Q8NCU7, P02750, P00739, P01023, O14791 |
| Menstrual blood | A0A0X9T0H6, A0A109PW74, A0A1L2BU40, A0A2U8J931, A0A2U8J9D3, D3DTX7, H0Y4U4, O00429, O15117, O15240, O43182, P01833, P07477, P35270, P49747, P62280, Q08211, Q13586, Q14012, Q16543, Q16775, Q16799, Q567U6, Q8N392, Q8WUQ7, Q92619, Q92882, Q99733, Q99988  Q9UDT6, Q9Y608, A0A024RAN2, A0A2S1CVU8, A0A384NL10, A8K9E4, B3EWG3, B4DRW4, B4DWS3, B7ZLF8, H0Y300, O15231, O60218, O60235, P01024, P01040, P01817, P02679, P04080, P04211, P05109, P05120, P05164, P06702, P09341, P0DJI8, P0DP04, P14923, P18510, P19876, P22307  P22528, P29373, P31947, P32320, P32926, P41218, P42830, P49913, P54108, P55774, P58062, P59666, Q01469, Q02413, Q03591, Q0ZCF9, Q15102, Q15847, Q5K634, Q641Q2, Q6N094, Q6P089, Q86YQ4, Q8NEZ4, Q8TBX8, Q9BPY8, Q9HCY8, Q9HD89, Q9NQ84, Q9NQT3, Q9NZT1, Q9P0K7, Q9UBC9, Q9UBQ5, Q9UIV8, Q9H3P7, P12429, Q9HDC9, Q9NVJ2, P02452, P08123, Q9H6S3, Q9Y383, P55145, Q14112, P02795, P37198, Q15428, Q9UHB9, P19971 |
| Cervical mucus | P01833, P19652, P23142, P01033, P80188, P01024, P54108, P04908, P07476, Q16777, Q96KK5, P04792, P20160, P05109, P04075 |
| Urine | P01009, P30101, P06733, P02768, P02774, P51884, P37173, O60635, P16070, P24821, P08311, Q5T8F0, O75144, O95998, P07355, P17813, P15924, P07996, Q08174, Q9UN70, Q14515, P25311 |
